# Structural Insights Into the Opening Mechanism of C1C2 Channelrhodopsin

**DOI:** 10.1021/jacs.4c15402

**Published:** 2024-12-16

**Authors:** Matthias Mulder, Songhwan Hwang, Matthias Broser, Steffen Brünle, Petr Skopintsev, Caspar Schattenberg, Christina Schnick, Sina Hartmann, Jonathan Church, Igor Schapiro, Florian Dworkowski, Tobias Weinert, Peter Hegemann, Han Sun, Jörg Standfuss

**Affiliations:** † PSI Center for Life Sciences, Laboratory for Biomolecular Research, Paul Scherrer Institut, Villigen 5232, Switzerland; ‡ Research Unit of Structural Chemistry & Computational Biophysics, Leibniz-Forschungsinstitut für Molekulare Pharmakologie, Berlin 13125, Germany; § Institute of Biology, Department of Experimental Biophysics, Humboldt-Universität zu Berlin, Berlin 10115, Germany; ∥ Institute of Chemistry, The Hebrew University of Jerusalem, Jerusalem 9190401, Israel; ⊥ PSI Center Photon Sciences, Laboratory for Femtochemistry, Paul Scherrer Institut, Villigen 5232, Switzerland; # Institute of Chemistry, Technische Universität Berlin, Berlin 10623, Germany

## Abstract

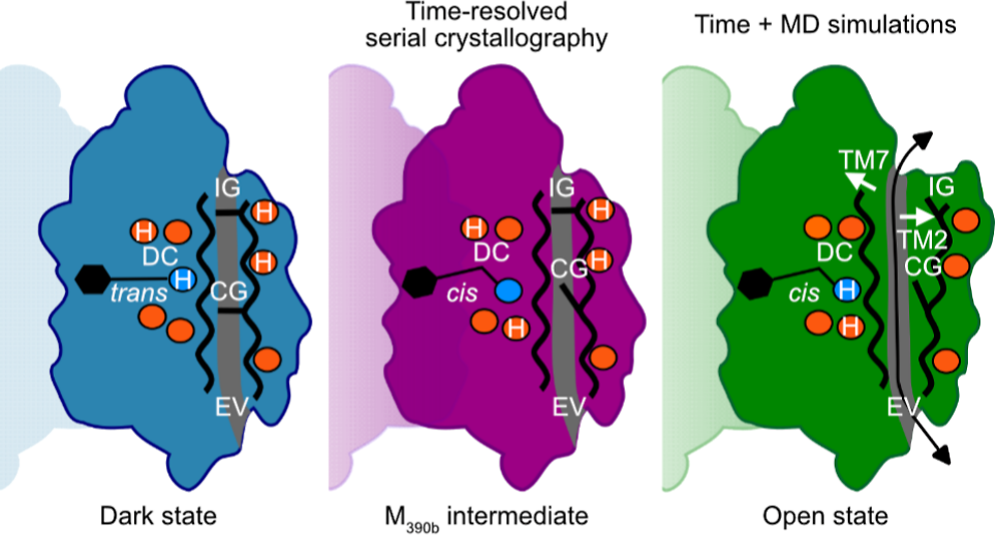

Channelrhodopsins, light-gated cation channels, enable precise control of neural cell depolarization or hyperpolarization with light in the field of optogenetics. This study integrates time-resolved serial crystallography and atomistic molecular dynamics (MD) simulations to resolve the structural changes during C1C2 channelrhodopsin activation. Our observations reveal that within the crystal environment, C1C2 predominantly remains in a light-activated state with characteristics of the M_390_ intermediate. Here, rearrangement of retinal within its binding pocket partially opens the central gate toward the extracellular vestibule. These structural changes initiate channel opening but were insufficient to allow K^+^ flow. Adjusting protonation states to represent the subsequent N_520_ intermediate in our MD simulations induced further conformational changes, including rearrangements of transmembrane helices 2 and 7, that opened the inner gate and the putative ion-translocation pathway. This allowed spontaneous cation conduction with low conductance, aligning with experimental findings. Our findings provide critical structural insights into key intermediates of the channel opening mechanism, enhancing our understanding of ion conduction and selectivity in channelrhodopsins at an atomistic level.

## Introduction

Channelrhodopsins are light-gated ion channels that allow mobile algae cells to find suitable conditions for photosynthesis. Beside their natural role as sensory photoreceptors, scientists have employed wild-type channelrhodopsins and their variants to control the depolarization of neural cells with light giving birth to the field of optogenetics. Targeting a genetically defined set of neurons can be used to understand their contribution to the behavior of animals^1^ or for medical applications like the partial restoration of vision in Retinitis Pigmentosa human patients.^2^ Optogenetic applications have sparked a wide interest into the study of channelrhodopsins to facilitate the design of improved variants with shifted absorption wavelengths,^3,4^ reduced desensitization,^5^ or modified ion conductance.^6,7^ Uncovering the molecular principles of how channelrhodopsins open in response to light is of high biophysical interest and may promote the design of additional optogenetic tools for neurobiology.

The photocycle of the most widely used optogenetic tool, channelrhodopsin-2 (ChR2) from *Chlamydomonas reinhardtii*, is well-known from extensive spectroscopic studies and could be related to time-resolved photocurrent measurements in cellular membranes.^1,8−10^ The activation cycle of dark-adapted ChR2 is initiated by the light-induced *trans*-to-*cis* isomerization of the *trans*,15-*anti* retinylidene chromophore into the first ground state K_520_ intermediate within 2 ps.^11^ This principle intermediate further relaxes into an L-type intermediate and a subsequent early M-species with deprotonated chromophore M_390a_. This intermediate, formed within 1 μs, provides the principal signal that initiates partial opening of the channel. However, primary channel opening occurs on a 100 μs time scale which is delayed compared to M_390a_ formation. This led to the suggestion that another conformational change compared to the M1 to M2 transition in bacteriorhodopsin (BR)^12^ is associated with the primary pore opening in a M_390a_-to-M_390b_ transition within about 100 μs.^10,13^ This M_390b_-state primarily conducts protons as shown by laser-stimulated electrophysiology.^10^ The fully open state with Na^+^ and K^+^ conductance is approached after 1 ms and contains a reprotonated chromophore named N_520_. Channel closing occurs on a 30 ms time scale under standard conditions and in detergent-solubilized protein, which however in cellular conditions strongly varies with extracellular and intracellular pH and membrane voltage. The LA_480_ state was originally assigned as a late photocycle intermediate is in reality a “light-adapted dark state”. It is a result of a C13=C14 and C15=N double isomerization into a 13-*cis*,15-*syn* species that only within minutes reconverts to the fully dark-adapted state.^10,14^ Photoactivation of the light-adapted dark state again initiates C13=C14 isomerization and a *syn*-cycle with all photointermediates in *syn*-configuration and a low conductance state with prolongated decay.

The first crystal structure of a channelrhodopsin has been solved for a chimera between Channelrhodopsin-1 and Channelrhodopsin-2 from *C. reinhardtii* commonly known as C1C2.^4^ This chimera with transmembrane helices (TM) 1–5 from ChR1 and 6–7 from ChR2 is more stable in detergent than the parental ChRs and provided the first invaluable insight into the overall architecture of the central ion conducting core.^4^ It shares the prototypical seven-transmembrane helical fold of microbial ion pumps and human G protein-coupled receptors. The structure further revealed a partial ion-conducting pathway reaching from the retinal binding pocket toward the extracellular side, lined by negatively charged residues suggested to secure cation selectivity. Very similar arrangements have been found in related structures of chrimson^15^ and channelrhodopsin-2.^16^ Indeed, some of these residues were later included in the structure-guided design of a light-gated chloride channel.^6^

Despite these successes it remains largely unknown how the channel opens at the atomistic scale due to lack of an open state structure. A first constriction of the putative channel, called the central gate which is composed of residues Glu129, Asn297 and Ser102, is found close to the retinal Schiff base and its counterion residues Glu162 and Asp292 (Channelrhodopsin-2 numbering can be obtained by subtracting 39 from the C1C2 residue numbers). A second ion constriction site, called the intracellular gate because of its location closer to the cytosolic site, is formed by Glu122, His173 and Tyr109. Both gates would need to open to connect the intracellular side with the vestibule on the extracellular side of the putative channel to allow full conductance. But the molecular details and sequence of events leading to the open state remain largely elusive.

Time-resolved serial crystallography is a powerful technique to study structural changes in light-activated proteins with up to femtosecond temporal and near-atomic spatial resolution.^17^ It has been employed on a number of rhodopsins,^18^ including C1C2 where it revealed early rearrangements in the retinal binding pocket.^19^ Here we report how we used serial synchrotron crystallographic methods, initially developed to resolve late photointermediates in BR,^20^ to study light-activation of C1C2 in the millisecond range. We present conformational movements after photoactivation in the retinal binding pocket, as well as in the adjacent gates and relate these results to channel activation mechanisms. Starting from this structural intermediate, we adjusted the protonation state of the titratable residues involved in channel opening and performed large-scale molecular dynamics (MD) simulations under different transmembrane potentials. These simulations further opened up the C1C2 channel, resulting in a fully open N_520_-like state that revealed a number of cation permeation events, with the simulated K^+^ conductance matching the experimental ranges. Our study provides structural insights into key intermediates of the channel opening during the photocycle, enabling the understanding of ion conduction and selectivity in channelrhodopsins at an atomistic scale.

## Results

### Light-Activated Structure of C1C2

**Figure 1:**
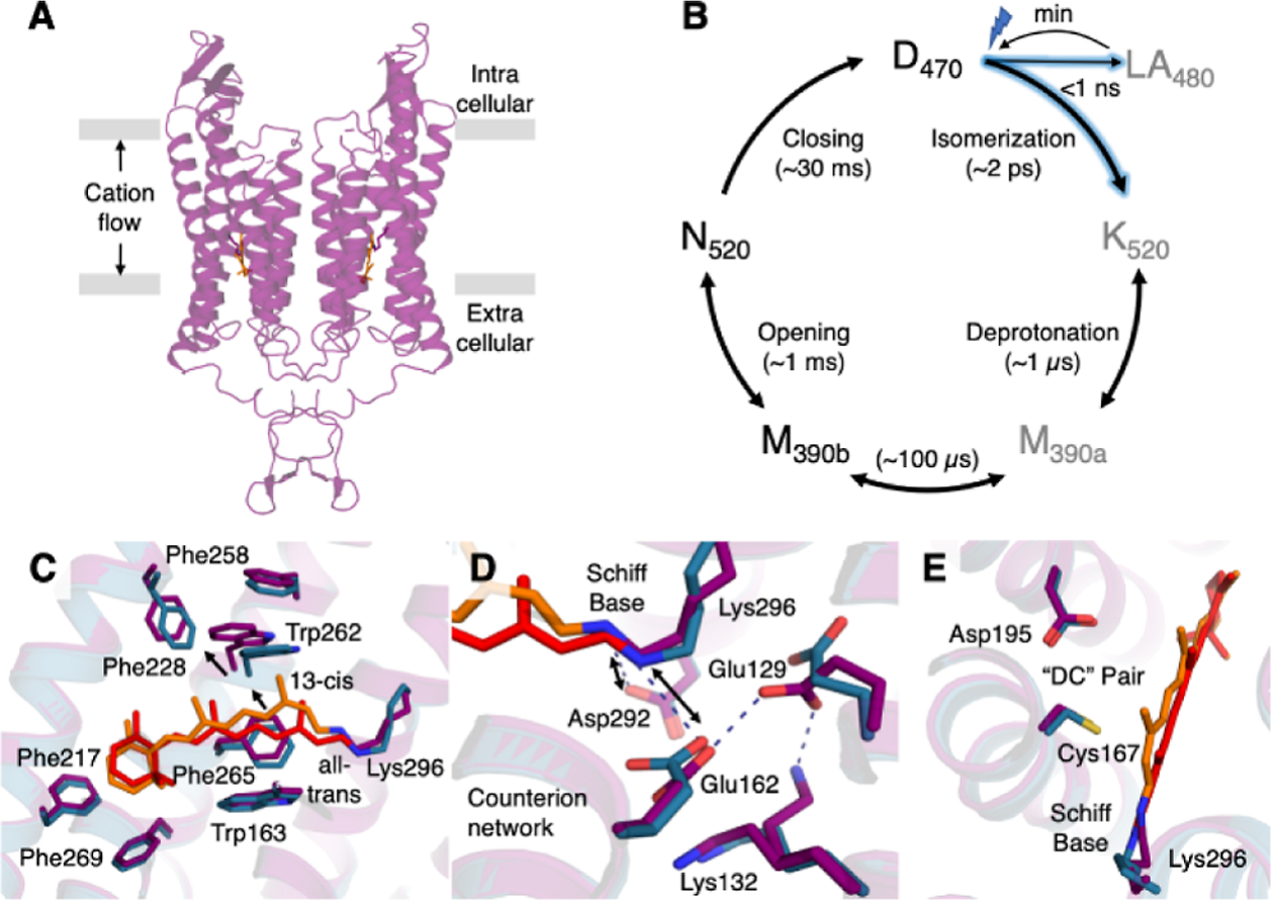
Light-activation of C1C2 channelrhodopsin. (A) Overview of the C1C2 dimer structure. The cellular membrane and the main flow of cations are indicated. (B) The channelrhodopsin photocycle is composed of several spectroscopic intermediates with characteristic absorption maxima that depend on the environment of the retinal chromophore. The first transition (D_470_-to-K_520_) is dominated by retinal isomerization, followed by Schiff base deprotonation (K_520_-to-M_390_) and reprotonation (M_390_-to-N_520_) during channel opening. Approximate times are shown based on spectroscopic studies in solution. (C) The light-activated structure shows how the binding pocket has adapted to retinal isomerization. (D) Local rearrangements in the counterion network indicate deprotonation of the retinal Schiff base without (E) rearrangements in the DC pair.

For our room temperature analysis of light-induced conformational changes in C1C2 we purified recombinant C1C2 from insect cells and grew crystals in lipidic mesophases following established conditions^4^ and subjected them to serial synchrotron crystallography^21^ at the Swiss Light Source, where they diffracted with anisotropic resolution up to 2.6 Å (Supporting Information, Table S1). An initial data set collected without illumination resulted in a dark state structure that is principally identical to earlier reports (root means square deviations of Cα atoms = 0.42 and 0.63 to 3UG9^4^ and 7C86,^19^ respectively). Next, we collected data while continuously illuminating the extruding lipidic cubic phase with the C1C2 crystals using a laser diode. The speed of extrusion was adjusted so that crystals were illuminated for approximately 100 ms before diffraction patterns were recorded. In comparison to other rhodopsins, retinal isomerization in C1C2 has a relatively low quantum efficiency around 30%^22^ and, in addition, single flash illumination of dark-adapted channelrhodopsins can yield a fraction of the protein in the desensitized light-adapted state.^10^ Together these effects likely resulted in the low activation levels in a previous time-resolved X-ray free electron laser study relying on nanosecond laser excitation.^19^ The millisecond exposure with our laser diode, even at the employed low laser fluence of 0.2 mJ/cm^2^, led to higher activation levels of 30% and excellent Fo(light)–Fo(dark) difference electron density maps (Supporting Information, Figure S1A). Data was of sufficient quality to calculate extrapolated structure factor amplitudes and to model structural changes upon illumination (Figure 1).

In order to further narrow the time where structural changes occur and to reduce the probability of dark-adapted to light-adapted conversion, we have illuminated crystals for 5 ms and collected X-ray data for 200 ms in bins of 5 ms. Pearson correlation analysis (based on^23^ with modifications described in^24^) of electron density changes over time (Supporting Information, Figure S1B) confirm accumulation of the light-activated intermediate within about 50 ms and its subsequent decay toward the dark state structure.

### Effect of Retinal Isomerization on the Overall C1C2 Structure

The initiating event in the photocycle of microbial rhodopsin is the all-*trans* to 13-*cis* isomerization of the retinylidene molecule. Beyond sharing this fundamental reaction, the conformations do vary between rhodopsins and can furthermore change throughout the photocycle, in BR, for example, from a twisted conformation in the K state toward a fully planarized retinal in the M intermediate.^25^ The retinal molecule in our structure of photoactivated C1C2 has fully isomerized into a planar 13-*cis* conformation that tilts the retinal polyene backbone toward the intracellular side (Figure 1C), as well as shifting the chromophore sidewards toward TM3 and TM4. Interestingly, this conformation is markedly different to those observed in the previous X-ray laser study of C1C2 activation^15^ and much closer to that of the BR M-intermediate^26^ (Supporting Information, Figure S2). The binding pocket in C1C2 is lined by aromatic residues (Trp163, Phe217, Trp262, Phe265 and Phe269) that hold the hydrophobic polyene backbone and the β-ionone ring of the retinal in place. Our light-activated structure shows major rearrangements in this hydrophobic pocket (Figure 1C). In particular the shift in Trp262, likely resulting from a steric clash with the C20 methyl group of the retinal, is very pronounced and transmits further changes toward the intracellular side of TM5 and TM6. Relocation of the β-ionone ring away from Phe269 and Phe217, together with the shift of the polyene toward Phe265 and away from Trp163 introduce further changes into the hydrophobic core of the protein. Remarkably, these substantial early rearrangements occur with only minor changes in the overall packing of the 7TM bundle (root-mean-square deviation of Cα = 0.4 Å), similar to what we observed in early intermediates of other rhodopsins.^24,25,27^

### Light-Activated Structure Represents an Early Deprotonated Intermediate

Beside retinal isomerization as the primary trigger of activation in all rhodopsins, the state of the retinylidene that links the retinal chromophore to TM7 is of particular functional relevance. Since channelrhodopsin photoactivation can lead to the formation of a 13-*cis*,15-*anti* retinylidene of the standard photocycle and with lower efficiency 13-*cis*,15-*syn* retinylidene of the light-adapted state both configurations have to be considered. While both configurations are structurally very similar, refinement using 13-*cis*,15-*anti* restrains yielded the overall better fit to the electron density map.

The second critical step in channelrhodopsin activation is controlled by the protonation state of the Schiff base, which deprotonates in the K_520_-to-M_390_ transition and is reprotonated in the M_390_-to-N_520_ transition.^28^ In C1C2, two negatively charged residues, Glu162 and Asp292, are close enough to act as counterion to stabilize protonation of the retinal Schiff base.^29^ Proton transfer from the Schiff base to Asp292 leads to a strong blue shift of absorption, the hallmark of the M_390_ intermediates which is required for the cation channel to open.^8^ In our light-activated C1C2 structure distances between the Schiff base and Glu162 and Asp292 have increased to 5.2 and 3.9 Å, respectively (Figure 1D). While we cannot observe protonation states directly, the breaking of these critical interactions suggests the Schiff base to be deprotonated in our light-activated structure.

A third step is enabled by reprotonation of the Schiff base during the M_390_-to-N_520_ transition with the proton coming most likely from a water around the Schiff base.^30^ Mutations in residues Asp195 and Cys167 strongly alter gating kinetics^31^ suggesting high relevance for the transition to the conductive state,^32,33^ even though the on-gating function of this pair has never been substantiated. In our light-activated structure the isomerized retinal is shifted toward this “DC pair” and a negative difference peak on Wat4 indicates that it has shifted from its position between Asp195 and Cys167. However, we do not observe the extreme shift of the retinal toward the “DC pair” suggested by previous TR-SFX^19^ experiments and computer simulations.^34^ In our light-activated structure, the “DC pair” remains basically in the same position (Figure 1E) as in the dark state, confirming that reprotonation of the Schiff base has not occurred yet.

**Figure 2:**
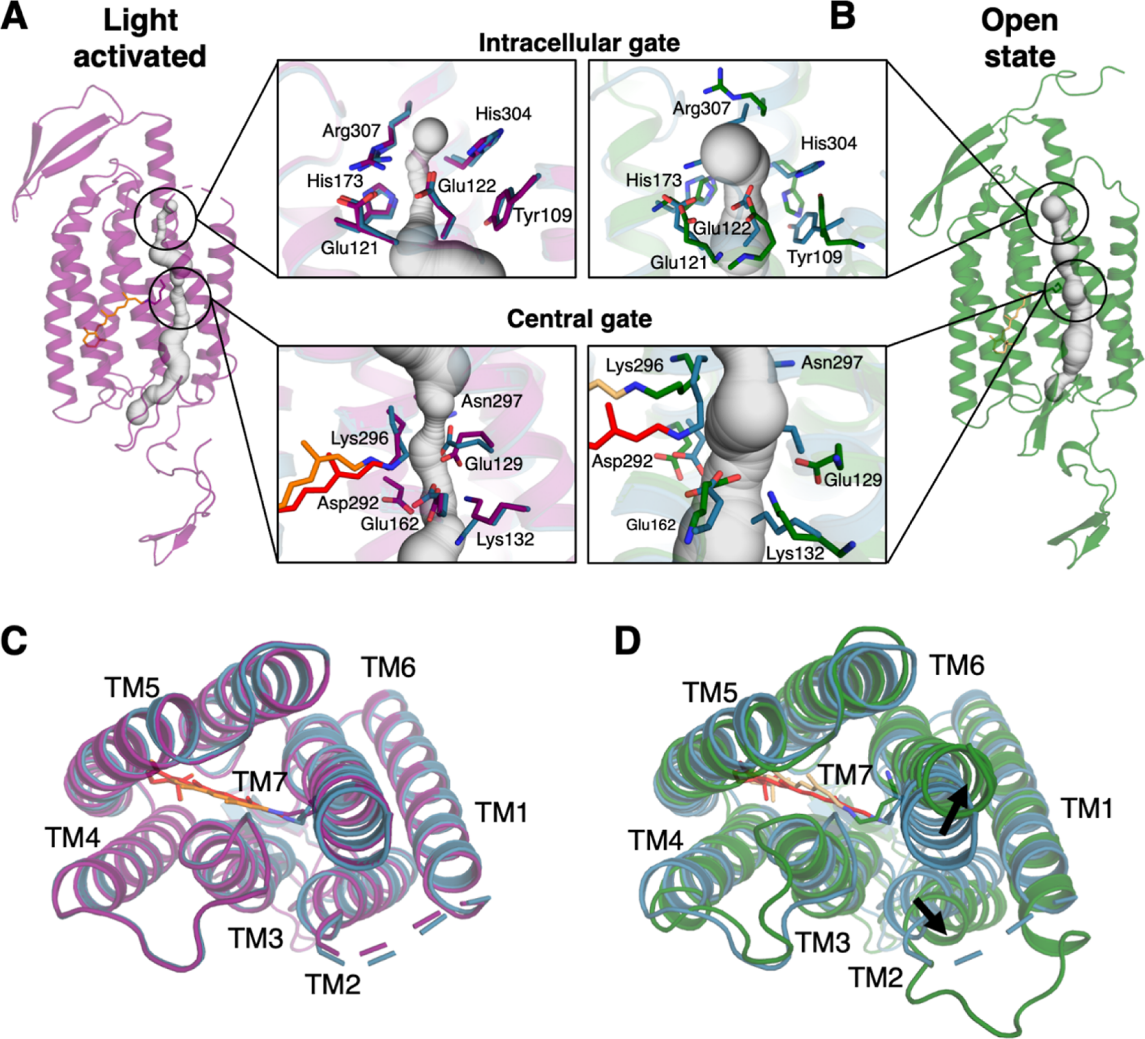
Opening the ion-conduction channel. (A) In the light-activated C1C2 structure (purple cartoon), conformational changes along the putative ion-translocation channel (gray) are predominantly located in the central gate (lower inset), leaving a bottleneck of 1.4 Å at the intracellular gate (upper inset). (B) Adjusting the protonation state based on spectroscopic characterizations of the open state (N_520_) leads to significant rearrangements in molecular dynamics simulations (right, green cartoon). The channel has further widened to a minimal width of 2.4 Å along the major bottlenecks sufficient for K^+^ ions translocation (compare Figure 3). The insets show a superposition of structures from the dark state (teal), light-activated state (purple), and open state (green), with key residues and the retinal chromophore depicted as sticks. (C) The overlay of the dark (teal) and light-activated (purple) C1C2 crystal structures indicates no large-scale rearrangements in the 7TM helical bundle. (D) In contrast the open state (green) is characterized by dominant shifts in TM2 and TM7 (black arrows) to open the cation conducting channel.

In concert with the changes in the retinal and Glu162, Lys132 adopts an alternative conformation and forms an ionic interaction with the central gate residue Glu129 (Figure 1D). Probing the structure with a sphere of 1.6 Å diameter placed into the extracellular vestibule results into a continuous channel through this constricting region toward the intracellular gate formed by Glu122, His173 and Tyr109 (Figure 2). These residues adopt principally the same positions as in our structure of the C1C2 dark state indicating, that this gate remains in a closed conformation. Based on these observations, we suggest our light-activated structure to represent a M_390_-like intermediate where the Schiff base is deprotonated and the channel is only partially opened and conductive only for protons. This assignment is in agreement with time-resolved spectroscopy on C1C2 crystals,^19^ where N_520_ formation is hindered and the M_390_ intermediate accumulates within milliseconds after photoactivation.

### Opening the Cation Conducting Channel

To elucidate the conformational changes required to fully open the channel, we performed atomistic molecular dynamics (MD) simulations starting from the light-activated structure. In one set of simulations, we deprotonated the retinal Schiff base while protonating Glu122, Glu129, Asp195, and Asp292 to represent the M_390b_ state. The second set of simulations was biased toward the open N_520_ conformation, with a protonated retinal Schiff base and Asp292, but deprotonated Glu122, Asp129, and Asp195. As a further control, we performed the same simulations starting from the dark state structure. The protonation states for the D_470_, M_390b_, and N_520_ states are shown in Figure 4 and summarized in Supporting Information, Table S2. These protonation states were defined based on a previous study using time-resolved Fourier transform infrared spectroscopy (FTIR) spectroscopy, which demonstrated that the deprotonation of Glu90 in ChR2 (Glu129 in C1C2) is crucial for pore opening.^35^ This finding is further supported with an integrated study combining electrophysiology analysis and MD simulations, showing that the deprotonation of two glutamate residues, Glu122 and Glu129, located at the intracellular and central gates, is critical for channel opening and water pore formation.^36^ Additionally, it has been concluded from several spectroscopic, electrophysiological measurements and computational simulations that Asp292 and rather than Glu162 is the proton acceptor during K/L to M transition.^30,32,36,37^ This conclusion aligns with our time-resolved M_390b_ structure, where the Schiff base is positioned closer to Asp292 than to Glu162. However, we are aware that controversies exist regarding the protonation and deprotonation of these key residues.^13^

**Figure 3:**
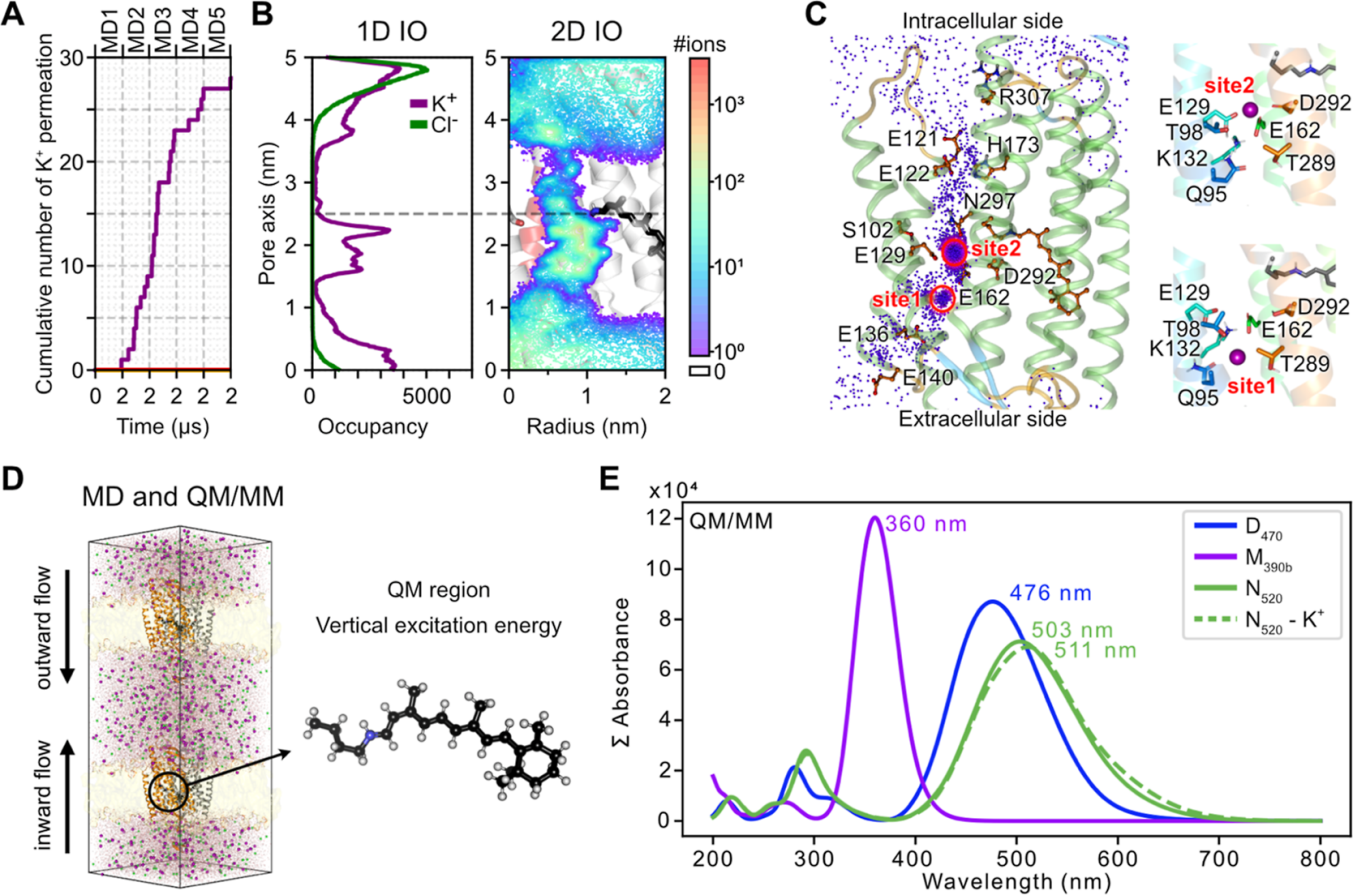
Simulated potassium ion conduction through the open C1C2 pore. (A) Cumulative number of inward K^+^ permeation events passing through the C1C2 pore (orange dark, red light-activated, purple open state). (B). (Left) One-dimensional K^+^ and Cl^–^ ion occupancy along the pore axis derived from the open state simulations. (Right) 2D projection of ion occupancy within the pore cylinder with a radius of 2 nm and a height of 5 nm centered at the Cα of Ser102. The protonated retinal Schiff base and Ser102 are represented as stick models. (C) (left) Cumulative K^+^ ions passing through the C1C2 pore, derived from MD simulations, are depicted as purple spheres and mapped onto the end snapshot. (Right) Two major K^+^ binding sites revealed by MD. (D) The system setup for computational electrophysiology and hybrid quantum mechanics/molecular mechanics (QM/MM) simulations. Dimeric C1C2 is represented as gray and orange cartoon models, with POPC shown as yellow surface. Water molecules, K^+^ ions, Cl^–^ ions are depicted as red and white sticks, purple spheres, and green spheres, respectively. A charge imbalance of 6 *e* resulted in the membrane potential of ±389 ±27 mV across the two compartments (inner and outer). The QM region for the hybrid QM/MM simulations and vertical excitation energy calculations is depicted as a ball-and-stick model: The Cα and Cβ bond of retinal Schiff base was cut and capped with a hydrogen link bond. (E) Averaged UV–vis spectra calculated from DFTB2 with dispersion correction. This excitation is on the ADC(2)/cc-pVDZ level of theory.

For each of these three distinct simulation setups, we embedded the channel into a palmitoyloleoylphosphatidylcholine (POPC) lipid bilayer, surrounded by 600 mM KCl, under transmembrane voltages of 300–500 mV (one C1C2 channel under positive voltage, the other one negative (Figure 3D). Then we conducted five independent runs of 2 μs each. Relatively high transmembrane voltages compared to physiological conditions were used to accelerate channel opening and ion conduction within the simulated time frame, a strategy extensively used in recent computational investigations of ion channels and membrane proteins.^38,39^ Our MD simulations showed system stability throughout the simulation period, as evidenced by the backbone RMSD plots (Supporting Information, Figure S3), which plateaued and remained less than 3 Å.

In both the dark-state and M_390b_ protonation state simulations, the channel remained closed with no K^+^ permeation events (Supporting Information, Figures S4 and S5). Even though the central gate and extracellular vestibule in the light-activated structure were more open compared to the dark-state structure, the changes were not sufficient to allow K^+^ permeation (Figure 2A). Overall, the simulations thus supported our assignment of the light-activated structure to a noncation-conducting early intermediate.

More significant conformational changes (root-mean-square deviation of backbone atoms in TM helices = 0.85–2.8 Å in the latter 1 μs of five 2 μs simulations) occurred in the set of simulations where we biased the structure from time-resolved serial crystallography toward the N_520_ intermediate by altering well-characterized protonation switches (Figure 2B, Supporting Information, Table S2). During these simulations, we observed substantial differences in the retinal binding pocket in both the central and intracellular gate (Supporting Information, Figure S6). Interestingly, both Trp262 and the interacting retinal chromophore, were considerably more dynamic in the open state simulations compared to the dark-state ones and the already significant conformational shift in the light-activated structure (Supporting Information, Figures S7 and S8).

These local changes are accompanied by a pronounced opening of the 7TM bundle compared to the dark and light-activated structures (Figure 2C,D). The most significant changes were observed in the intracellular regions of TM1, TM2, TM6, and TM7, as well as the extracellular region of TM2, as evidenced by the per-residue backbone deviations calculated at different time frames of the MD simulations starting from the light-activated structure (Supporting Information, Figure S9). The conformational change in the opening of C1C2 notably differs from that reported in a recent study of the anion channelrhodopsin GtARC1.^40^ Based on QM/MM and MD simulations, this study predicted a more localized conformational change around the retinal, leading to the opening of the constriction site in the ion permeation pathway. In contrast, the magnitude of changes in C1C2 are more akin to those observed during the formation of the open N-state structure of BR, where rearrangements in TM5, TM6 and TM7 initiate reloading of the proton pump.^20^

In C1C2 these changes are necessary for ion conduction and accordingly we observed 28 continuous K^+^ inward permeation events during the open state simulations (Figure 3A). This corresponds to a very low conductance of 1.2 ± 1.1 pS, which falls within the range of experimentally estimated single-channel conductance.^41^ Strong inward rectification was observed in the simulations, with very low outward conductance (Supporting Information, Figure S10), which aligns well with the electrophysiology data.^42^ Furthermore, our simulations revealed strict cation selectivity, with strong K^+^ binding but no Cl^–^ binding within the channel pore, likely due to the strongly electronegative environment (Figure 3B, Supporting Information, Figure S11). Moreover, we identified two neighboring stable K^+^ binding sites on the extracellular side of the channel pore, close to the central gate. At site 1, K^+^ is coordinated by Glu162, Thr289, Gln95, and Thr98, while at site 2, K^+^ is mainly coordinated by Glu162, Glu129, and Asp292 (Figure 3C). During conduction, K^+^ density within the pore was very low, with only one K^+^ ion conducted through the pore most of the time (Supporting Information, Movie S1). This conduction mechanism differs from those observed in most other cation channels, such as K^+^, Na^+^, and several nonselective cation channels.^38,43^ These channels are multi-ion, single-file pores, where multiple ions permeate through this narrow region via a water-mediated or direct knock-on mechanism. In contrast, in Chlamydomonas the photocurrent is graded over 4 orders of magnitude in light intensity^44^ which requires channelrhodopsin to function as a one-ion pore, operating by simple mutual exclusion and a very low conductance.

As a further step, we evaluated the different intermediates obtained from time-resolved serial crystallography and MD by predicting the vertical excitation energy using a hybrid quantum-mechanical molecular mechanics (QM/MM) approach (Figure 3E). The ground state QM/MM calculations were carried out with the AMBER package version 20,^45^ where the QM region was treated using the DFTB2 or DFTB3 tight-binding methods (computational details are provided in the Materials and Methods section in the Supporting Information). DFTB is a semiempirical method, but typically provides a reasonable compromise between performance and accuracy. Similar computational protocols were successfully applied in previous studies in the field (see for example ref (46)). Using the production runs from the QM/MM MD simulations, absorption spectra were subsequently calculated at the RI-ADC(2)/cc-pVDZ^47^ level of theory with the Turbomole program suit version 7.7.^49^ The predicted maximum absorption of the light-activated state with deprotonated retinal was 360 nm slightly lower than experimentally observed difference of 390 nm but both are similarly blue-shifted compared to the dark-state. Furthermore, the MD-predicted open state showed a λ_max_ of 503 nm when no K^+^ is bound at site 2 and 511 nm when one K^+^ is bound at site 2. The difference between the open state and dark state is in good agreement with the experimentally characterized differences in the maximum absorption.

It should be noted that our MD simulations are conceptually different from a previous MD investigation, which characterized key intermediates by analyzing the number of water molecules in the central gate region and interpretating the potential mean force profiles using an enhanced sampling approach.^48^ These earlier simulations were unable to ensure that the channel reached a fully conductive state with spontaneous ion permeation events that could be directly compared to electrophysiology data.

## Conclusions

In our study, we combined time-resolved serial crystallography with atomistic MD simulations to elucidate the major structural events during C1C2 channelrhodopsin activation. Within the crystal environment, C1C2 remained in a light-activated state that we assigned to an M_390_-like intermediate, where changes in the retinal binding pocket partially opened the central gate toward the extracellular vestibule.

**Figure 4:**
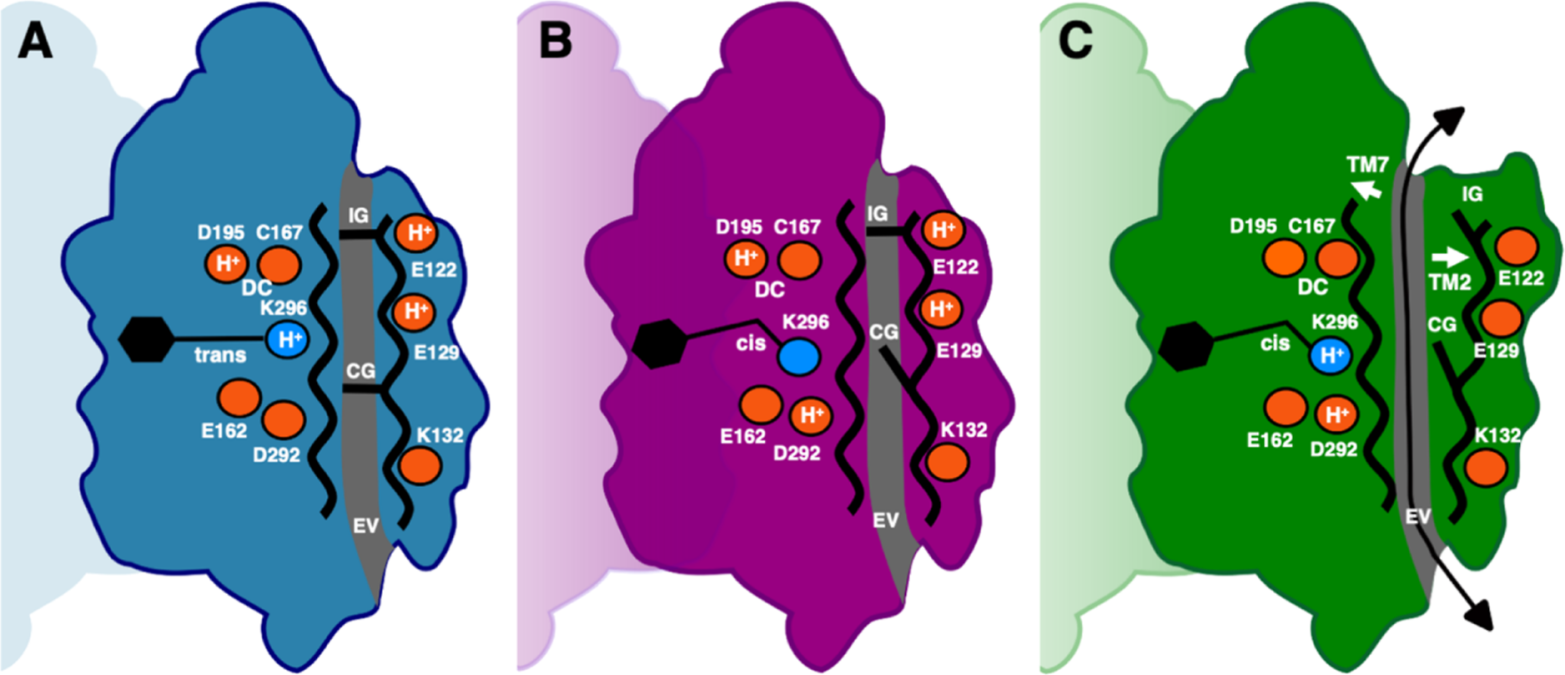
Schematic representation of the C1C2 opening mechanism. (A) In the dark state, (B) the M_390b_ intermediate and (C) the N_520_ intermediate. The retinal is shown in black and key residues discussed in the main text are shown as small rings. Protonation states (H^+^) used during the MD simulations are shown. The intracellular gate (IG), the central gate (CG) and the extracellular vestibule (EV) along the putative ion conduction channel (gray) are indicated. Opening of the channel is accompanied by rearrangements of the seven-transmembrane helical bundle and in particular of TM2 and TM7 (white arrows).

While these structural changes widened the putative channel through the protein and may conduct H^+^, they were not sufficient to enable the flow of K^+^ in our simulations. Only when we set protonation switches in the central and intracellular gates, and DC pair to represent the N_520_ intermediate did we observe conformational changes that opened the intracellular gate, establishing a full channel for alkali cation but not anion conductance. The necessary structural changes can be illustrated by a morph between the light-activated structure and the simulated open state (Supporting Information, Movie S2). The mechanism, where initial light-induced changes partially open the channel but further protonation changes and larger helix arrangements and inner gate opening are required for the full conductance (Figure 4), highlights the complex nature of channelrhodopsin activation.

The general agreement between the simulated cation conductance and electrophysiology data, together with the absorption spectra calculated from our structural intermediates, suggests that this series of events closely resembles the structural changes upon channel opening. Further investigations could focus on characterizing additional intermediate states in the photocycle using a combination of time-resolved serial crystallography and computational simulations. MD simulations performed under transmembrane voltages would be particularly useful for providing atomistic insights into voltage-dependent ion binding and competition—an important characteristic of many channelrhodopsins.^42^ Furthermore, increasing K^+^, Na^+^, and Ca^2+^ selectivity in channelrhodopsins remains a cutting-edge research direction, as these improved optogenetic tools would enable more precise control over neuronal firing and synaptic activity, as well as facilitate the study of calcium-dependent processes with high precision. The atomistic insights gained from this study, along with the methods developed, could pave the way for the rational design of next-generation channelrhodopsins with enhanced ion selectivity for optogenetic applications.

## Data Availability

Coordinates and structure factors have been deposited in the PDB database under accession codes 9GO1 (for the C1C2 dark state) and 9GO2 (for the light-activated state). Data relevant to molecular dynamic simulations has been deposited in Zenodo linked under https://doi.org/10.5281/zenodo.14245539.

## Supporting Information Available

During conduction, K^+^ density within the pore was very low, with only one K^+^ ion conducted through the pore most of the time (MP4)
The necessary structural changes can be illustrated by a morph between the light-activated structure and the simulated open state (MP4)
Materials, methods, and additional experimental and calculation results are included in the Supporting Information (PDF)
