# Supplementary material for: Structural Insights Into the Opening Mechanism of C1C2 Channelrhodopsin
Source: J Am Chem Soc. 2024 Dec 16;147(1):1282–90. doi: 10.1021/jacs.4c15402 (PMC11726564; doi:10.1021/jacs.4c15402)
Supplement: Supplementary file 3 — ja4c15402_si_003.pdf [file ja4c15402_si_003.pdf]

Supporting information for

Structural insights into the opening mechanism of C1C2 channelrhodopsin

Matthias Mulder[1,‡], Songhwan Hwang[2,3,‡], Matthias Broser[3], Steffen Brünle[1], Petr Skopintsev[1], Caspar Schattenberg[2], Christina Schnick[3], Sina Hartmann[1], Jonathan Church[4], Igor Schapiro[4], Florian Dworkowski[5], Tobias Weinert[1], Peter Hegemann[3], Han Sun[2,6,*], Jörg Standfuss[1,*]

[1] PSI Center for Life Sciences, Laboratory for Biomolecular Research, Paul Scherrer Institut, 5232 Villigen, Switzerland.

[2] Research Unit of Structural Chemistry & Computational Biophysics, Leibniz-Forschungsinstitut für Molekulare Pharmakologie, Berlin 13125, Germany.

[3] Institute of Biology, Department of Experimental Biophysics, Humboldt-Universität zu Berlin, Berlin 10115, Germany.

[4] Institute for Chemistry, The Hebrew University of Jerusalem, Jerusalem, Israel

[5] PSI Center Photon Sciences, Laboratory for Femtochemistry, Paul Scherrer Institut, 5232 Villigen, Switzerland.

[6] Institute of Chemistry, Technische Universität Berlin, Berlin 10623, Germany.

‡ authors contributed equally

* to whom correspondence should be addressed

Materials and Methods

Protein expression and purification

The human codon-optimized sequence of chimeric channelrhodopsin C1C2[1], including a C-terminal tobacco etch virus (TEV) protease cleavage site followed by an octa-histidine tag, was cloned into the pFastBac1 vector (Invitrogen, Thermo Fisher Scientific, Waltham, MA, USA) using BamH1 and HindIII restriction sites. Protein expression in insect cells was carried out according to the Bac-to-Bac method, with bacmid preparation, transfection, virus production, and expression in Sf21 cells (Gibco, Thermo Fisher Scientific, Waltham, MA, USA), as described previously [2]. After harvesting the cells all subsequent purification steps were performed at 4°C under red light. Cells were solubilized for 4h in buffer A (20 mM Tris-HCl pH 8.0, 100 mM NaCl) supplemented with 2% n-dodecyl-β-D-maltoside (DDM, Glycon Biochemicals GmbH, Luckenwalde, Germany), 0.4% Cholesterol hemisuccinate (CHS, Sigma-Aldrich, St. Louis, MO, USA), 5 µM all-trans retinal (Sigma-Aldrich) and complete protease inhibitor (Roche, Basel, Switzerland), and subsequently centrifuged at 40.000rpm for 30 minutes. The solubilized protein in the supernatant was purified by Ni^{2+} affinity chromatography (5ml HisTrap crude column (Cytiva, Marlborough, MA, USA)) followed by size-exclusion chromatography in buffer A with 0.05% DDM, 0.01% CHS (HiLoad 16/600 Superdex 200 pg (Cytiva)). Peak fractions with an absorption ratio of ~ 2 at 280/475 nm were pooled, concentrated using an Amicon Ultra Centrifugal Filter with a molecular weight cutoff (MWCO) of 100 kDa (Merck Millipore, Burlington, MA, USA) and flash frozen in liquid nitrogen.

Crystallization

We used a similar protocol for the crystallization of C1C2 as reported in[1]. In short, C1C2 was mixed with monoolein in a 2:3 protein to lipid ratio (w/w), in a 100 µL Hamilton syringe. The protein-LCP mixture was injected into crystallization buffer, containing 100 mM sodium citrate (pH 6.0), 30% PEG500DME, 100 mM $MgCl_2$, 100 mM NaCl, and 100 mM $(NH_4)_2SO_4$. Crystals were grown in 2-3 weeks in the dark at 20 °C. Crystal-laden LCP was pooled in a 500 µL Hamilton syringe with a small amount of crystallization buffer.

Serial data collection

Dark and light-activated data were collected at the PXI-X06SA beamline at the Swiss Light Source with the setup described in[3]. To prepare samples for serial crystallography, the crystal-laden LCP was mixed with monoolein to reach the right consistency for extrusion. Next, the crystal-laden LCP was injected in the intersecting X-ray and laser diode paths by a high viscosity injector[4] with a 50 µm diameter nozzle at a speed of 500 µm/s. Data was recorded on an EIGER 4M detector with a frame rate of 50 Hz. The X-ray beam had a size of 5x15 μm^2 at an energy of 12.4 keV. A 445 nm laser diode was used for illumination of the crystals with a spot size of 80 x 120 µm, with a laser power of 4.9 mW/mm^2, and an exposure time of about 100 ms. The time-resolved data was collected using a detector frame rate of 200 Hz using 200 ms cycle consisting of a 5 ms activation pulse from the laser followed by 39 bins of data.

Serial data processing and refinement

Peak finding and indexing were performed with CrystFEL[5], version 0.10.2, using the following settings: --indexing=xgandalf --peaks=peakfinder8 --threshold=10 --int-radius=4,5,7 --min-snr=3.5 –min-peaks=10 --min-pix-count=2 --min-res=80 –tolerance=5,5,5,3.0. Data was merged and scaled using partialator with model xsphere. The staraniso server was used for anisotropic correction of obtained data[6]. Isomorphous difference maps and extrapolated maps were calculated with Xtrapol8[7], using k-weighting, and were used to aid refinement of the light structure. Structural refinements were done using Phenix[8], version 1.20-4459, employing iterative cycles of manual adjustments made in Coot[9]. For final refinement of the light model the model refined against extrapolated data was combined with the dark model with an occupancy of 0.3 and 0.7 respectively and only B-factor and TLS refinement was carried out. All figures were created using the PyMOL Molecular Graphics System, Version 3.0 Schrödinger, LLC.

Identifying channels in C1C2

Channels in the structures were identified using the Caver web tool[10]. Caver identified a pocket around the extracellular vestibule that acted as the starting point for the channel, with coordinates: x=3.7, y=20.2, and z=13. The settings for caver to identify the channel in the light-activated structure were: minimum probe radius = 0.7, shell depth = 4, shell radius = 3, clustering threshold = 3.5, maximal distance = 3, and desired radius = 5. We chose a minimum probe radius of 0.7 to showcase the bottleneck around the central gate as this is too small for alkaline cations to pass, as

illustrated in **Figure 3**. For the simulated open structure, we changed the minimum probe radius to 0.8, the smallest size that allows alkaline cations to pass.

Molecular dynamics (MD) simulations

We prepared systems for MD simulation of the dark-state (PDB entry 7C86) and our M-like structure (PDB entry 9GO2) of C1C2 using CHARMM-GUI[11] with varied protonation states (**Supplementary Table 2**). For both the dark and open states, the missing region from Q112 to T117, as well as G329 and G330 was modeled using MODELLER 10.[12]. The dimeric C1C2 was embedded into 1-palmitoyl-2-oleoyl-sn-glycero-3-phosphocholine (POPC) and solvated with TIP3P water model[13] and 600 mM KCl. Retinal force field parameters from the published works[14] were used. CHARMM36m[15] was employed for the simulations. The prepared systems (**Supplementary Table 3**) were energy minimized using the steepest descent method and equilibrated in an isothermal-isobaric (NPT) ensemble with a stepwise release of positional and dihedral angular restraints on the backbone, side-chains of the protein, and POPC (**Supplementary Table 4**). To maintain a constant temperature at 303 K and pressure at 1 bar during the equilibrations, we used the velocity-rescaling thermostat[16] and Berendsen or Parrinello-Rahman barostats[17]. For the short-range van der Waals and Coulomb interactions, a cutoff of 1.2 nm was set. The particle mesh Ewald (PME) method[18] was used for long-range electrostatic interactions. The LINCS algorithm[19] was used to constrain the bonds between hydrogens and heavy-atoms. For the preparation of a system for MD-based computational electrophysiology (CompEL) simulations[20] a copy of the equilibrated system was stacked along the channel pore axis, resulting in a double lipid bilayer system. This system was energy minimized, followed by production simulations for 2 μs with two charge imbalances (4 *e* and 6 *e*). The resulting transmembrane voltages of each simulation setup are summarized in **Supplementary Table 5**. The boundaries of the two compartments, inner and outer, were defined by the center of mass of each group of Cα atoms of the central gate residues (Ser102, Glu129, and Asn297) of the dimer. To maintain a constant charge imbalance across the membranes, ions crossing a cylinder centered at the defined group with radius of 3 nm and an upper and lower height of 1.5 nm and 1 nm, respectively, were counted. Upon detection of a crossed ion, an ion of the same species in one compartment was exchanged with water in the other compartment using the CompEL algorithm. These simulations

were individually replicated five times. All MD simulations were conducted using GROMACS 2019.3 and 2019.6[21].

Analysis of the MD simulations

The plots of ion track (tracking the z-position of ions during their permeation), 1D and 2D ion occupancy were calculated using MDAnalysis 2.7[22], Matplotlib 3.8.4[23], Scipy 1.13[24] and Numpy 1.26.1[25]. To track ions in the ion conduction pathway, ions within a virtual cylinder centered at the Cα of Ser102 with a radius of 2 nm and a height of 5 nm were considered. The protein was centered in the box, removing the periodic boundary effects, and then fitted to the backbone atoms in the initial snapshot for analysis. Molecular visualization was conducted using PyMOL 3.0.3 (http://www.pymol.org/pymol) and VMD 1.9.4[26]. For the deposition of the open state MD snapshot, we selected a representative structure from the third replica, which showed the largest conductance under a membrane potential of -389±27 mV. This selection was based on a conformational clustering using the *gmx cluster* module of GROMACS[21], with a cutoff of 0.35 nm, resulting in 12 clusters. We chose the representative structure from the first cluster.

Hybrid quantum mechanics / molecular mechanics (QM/MM) simulations

For the dark, light-activated, and open state, one trajectory of the MD runs was processed into 100 equidistantly chosen snapshots. For the light-activated state, five input structures for the QM/MM runs were selected from these snapshots. As the channel opening is of predominant interest, we processed more trajectories within the dark and open state characterization. For the dark state, eight trajectories were processed, while for the open state, the snapshots were divided into those with and without a potassium ion in close vicinity to the retinal chromophore (potassium ions were considered close, if they were found in a 6.5 Å distance of retinal Schiff base, Glu162, and Asp292). Again, eight structures of each subset were included in the QM/MM runs. The selected snapshots were chosen from the entire shift range of the optimized structures (see below).

All QM/MM simulations were performed using the AMBER suit of programs, version 20[27]. Throughout the hybrid QM/MM simulations, the QM region was treated either by the DFTB2 or DFTB3 tight binding model as implemented internally in AMBER[28], using the MIO-L and 3OB parameter sets, respectively[29]. For DFTB2 runs, the implemented dispersion model was employed (DFTB2+D)[30].

The QM region of the QM/MM runs included the retinal Schiff base, capped with hydrogen at the Cα atoms and embedded in a point charge environment, i.e. electrostatic embedding was used. All non-QM atoms were described with the CHARMM36m all atom force-field[15] in all QM/MM simulations.

QM/MM runs are based on MD equilibrated snapshot structures, followed by QM/MM minimization with a short pre-minimization (250 steps; steepest decent algorithm) using constraints on the protein and lipids, followed by 250 steps of steepest descent and additionally 750 steps using the conjugate gradient method without any constraints on DFTB3 level of theory. The 100 ps equilibration was separated into a 50 ps heating step (100 K to 303 K) followed by 50 ps unconstrained equilibration. For each input structure, a production of 100 ps was performed. Equilibration and production runs were recorded predominantly on DFTB2+D level of theory. As a comparison and method validation for a subset of the data, equilibration and production was also performed on DFTB3 level of theory (**Supplementary Figure 12**). Since both computations lead to qualitatively similar results, we finally decided on DFTB2+D in order to benefit from the implemented dispersion correction.

Spectra were generated from equidistantly spaced snapshots (every 1 ps) of the production runs. In all cases, the retinal chromophore was extracted as the QM region, keeping the protein environment as a point charge embedding in the QM calculations. Spectra were calculated on RI-ADC(2)/cc-pVDZ[31] level of theory with the Turbomole programsuit version 7.7 (TURBOMOLE V7.7 2022, a development of University of Karlsruhe and Forschungszentrum Karlsruhe GmbH, 1989-2007, TURBOMOLE GmbH, since 2007.). The convergence of the self-consistent field (SCF) computations was set to 10^{-8}, additionally ensuring converged results by enforcing changes of the density matrix to be smaller than 10^{-7}. For SCF and ADC(2) excitation computations, the resolution of the identity approximation was used. Except for rare cases where convergence issues appeared (here the number of recorded excitations was throughout reduced to 8; 11 appearances in all calculations), the 10 lowest excited states were recorded. Spectra were computed as an average of all excitations and intensities were calculated from the oscillator strength of the individual excitations.

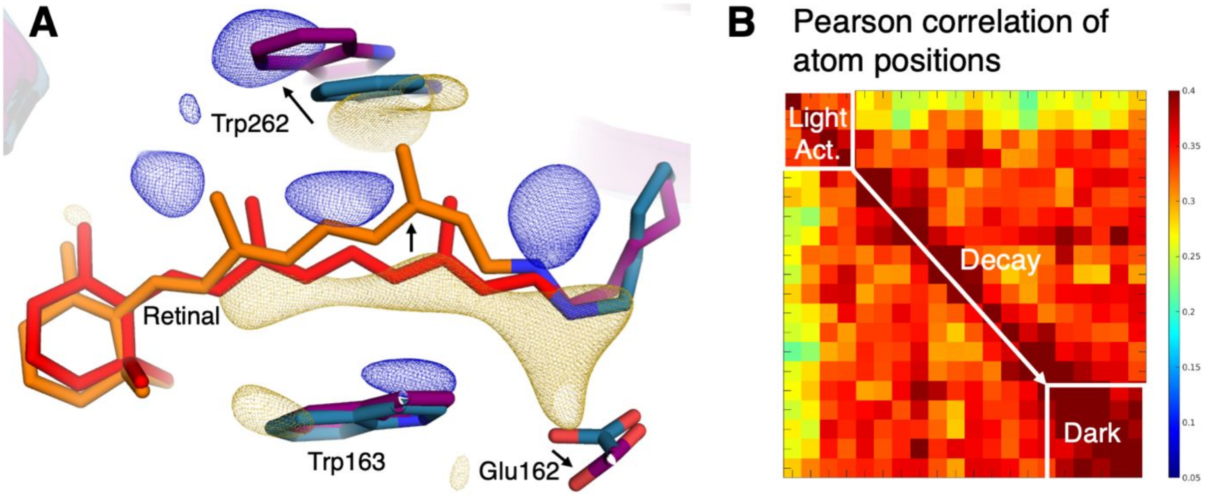

Supplementary Figure 1: Difference electron density map and Pearson correlation of atom positions. (**A**) View of changes within the retinal binding pocket. Movements of the retinal chromophore (red dark state, orange light-activated state) and nearby residues (dark in teal, light-activated in purple) are indicated by arrows. The experimental difference electron density map (Fo(light)-Fo(dark), gold negative, blue positive, contoured at 3.5 sigma) from the serial crystallographic experiment is shown. (**B**) Additional time-resolved serial crystallographic data was collected as described in[3] and binned into 10 ms intervals. Structural changes can be followed by the rmsd correlation of all atoms, including the retinal, in each binned structure. The results identify two major states at about 5-40 ms and 40-200 ms corresponding to the formation and decay of the light-activated structure.

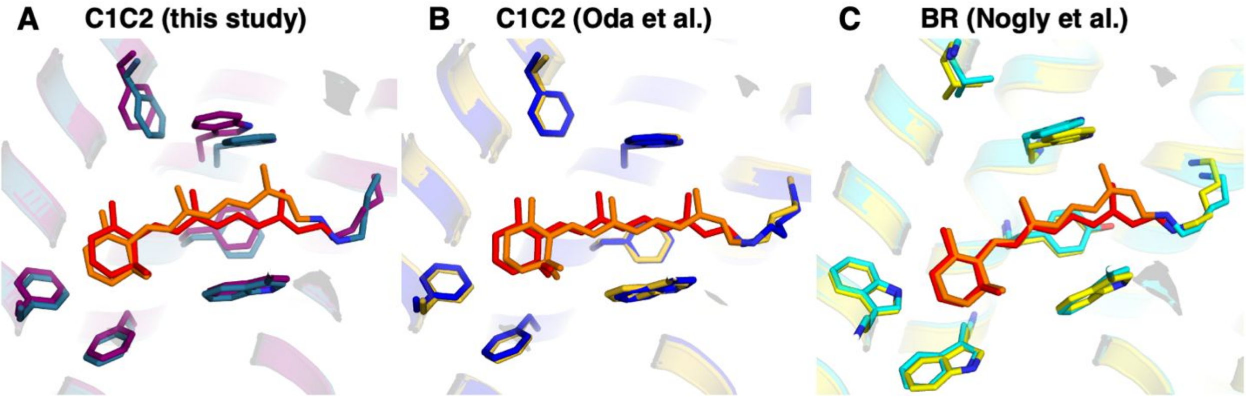

Supplementary Figure 2: Comparison of light-activated rhodopsin structures. (**A**) Structures of C1C2 channelrhodopsin from this study (red dark, orange light-activated for about 100 ms). (**B**) Structures of C1C2 taken from[32] (red dark, orange 4 ms after light-activation). (**C**) Structures of the BR taken from[33] (red dark, orange 8.33 ms after light-activation). It is interesting to note how similar our early deprotonated structure of C1C2 is to the M-intermediate structure of BR. In contrast our structure is markedly different to the C1C2 structures reported by Oda et al., who refined their data against a 13-cis,15-syn retinylidene conformation and very limited changes in the retinal binding pocket.

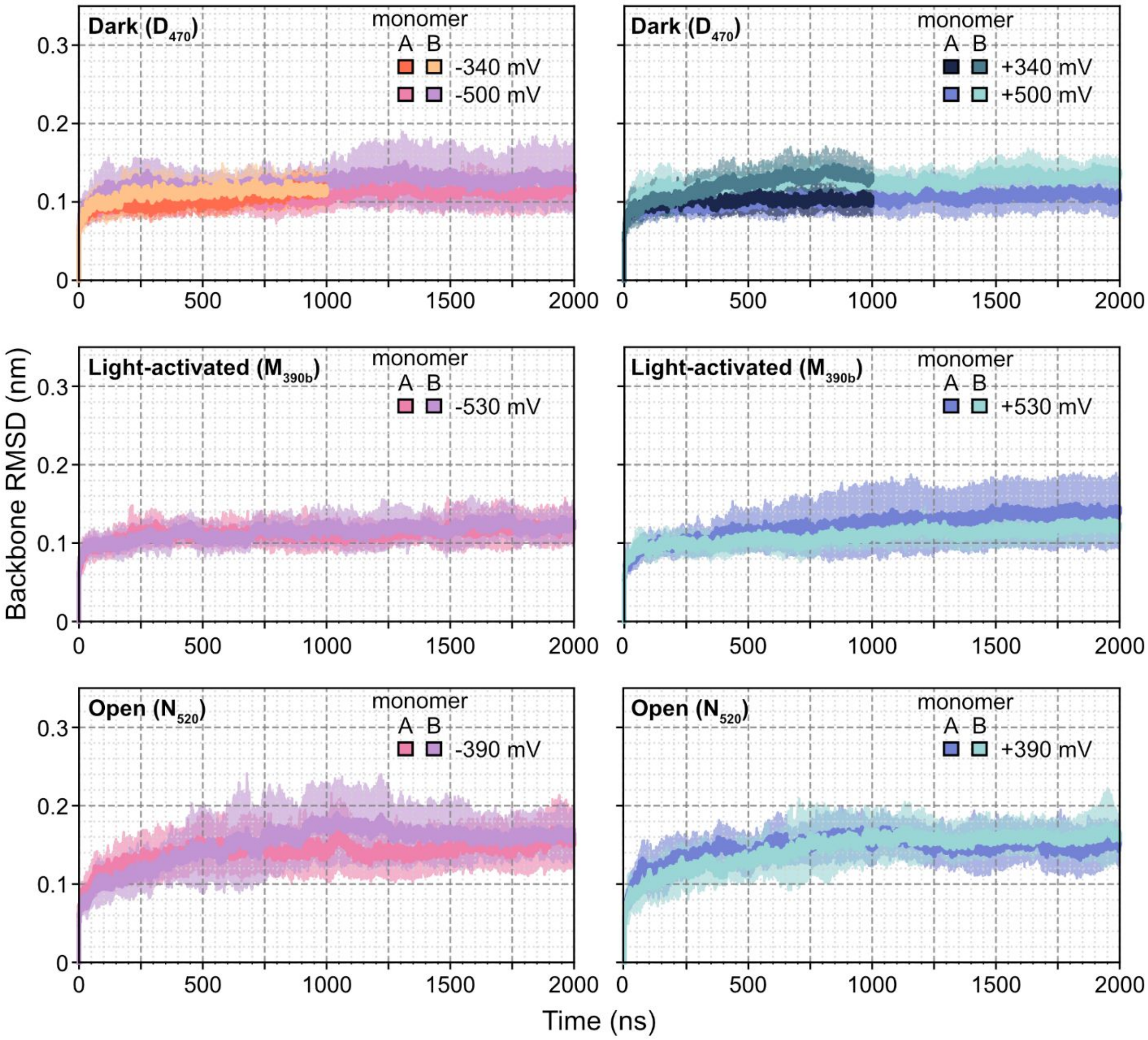

Supplementary Figure 3: Root-mean-square-deviations (RMSDs) during molecular dynamic simulations. The average RMSDs of backbone of each monomer in the dimeric C1C2 in the dark state, light-activated, and open state are shown as solid lines, while the standard deviations are shown as shaded area. For these RMSD calculations, residues 85 to 308 were considered, excluding the flexible extracellular amino and intracellular carboxyl termini. The simulations were conducted for 2 µs, except for the dark state at ±340 mV, which was run for 1 µs. Each simulation was replicated five times at 303 K and with a 600 mM KCl concentration.

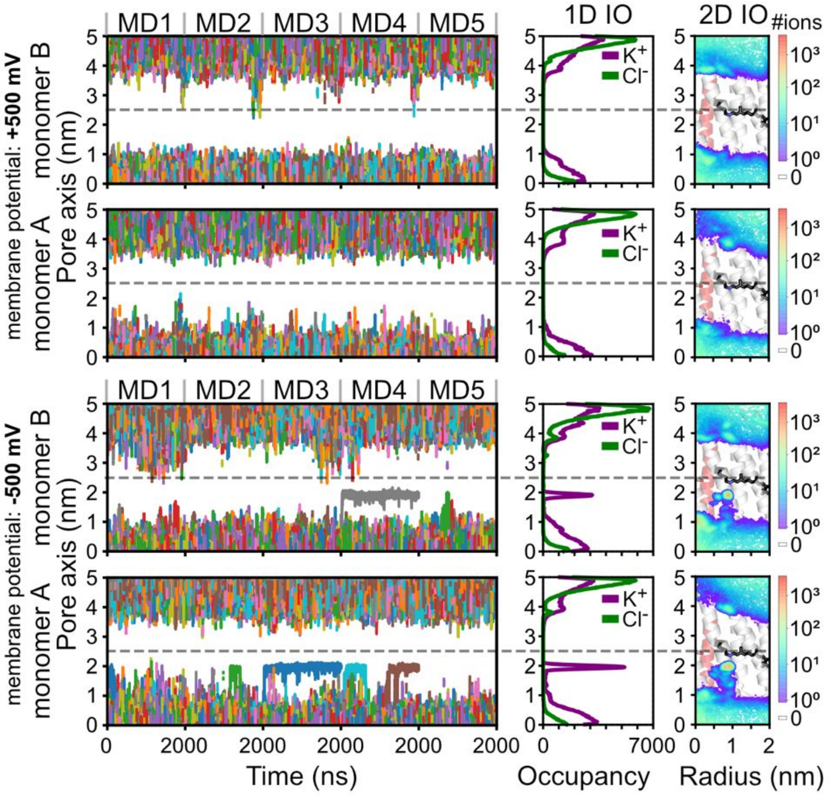

Supplementary Figure 4: Cation permeation in the dark-state of C1C2. (**left**) Traces of K^+ in the pore of C1C2 with the central gate highlighted as a dashed grey line. (**middle**) Cumulative 1D ion occupancy along the pore axis. (**right**) 2D K^+ density within the pore region, mapped onto the closed-state C1C2, with protonated retinal Schiff base and Ser102 depicted as stick models. The 2-µs simulations were replicated five times at 303 K with a 600 mM KCl concentration. No ion permeation events were observed in these simulations.

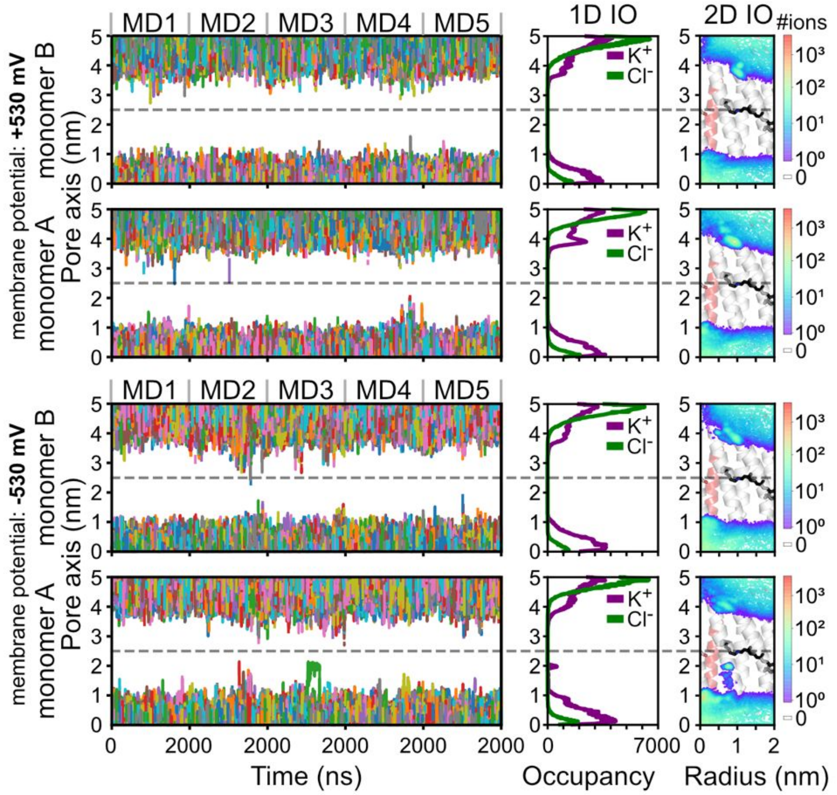

Supplementary Figure 5: Cation conduction through the light-activated C1C2 structure. (**left**) Traces of K^+ in the pore of C1C2 with the central gate highlighted as a dashed grey line. (**middle**) Cumulative 1D ion occupancy along the pore axis. (**right**) 2D K^+ density within the pore region, mapped onto the closed-state C1C2, with protonated retinal Schiff base and Ser102 depicted as stick models. The 2-μs simulations were replicated five times at 303 K with a 600 mM KCl concentration. No ion permeation events were observed in these simulations.

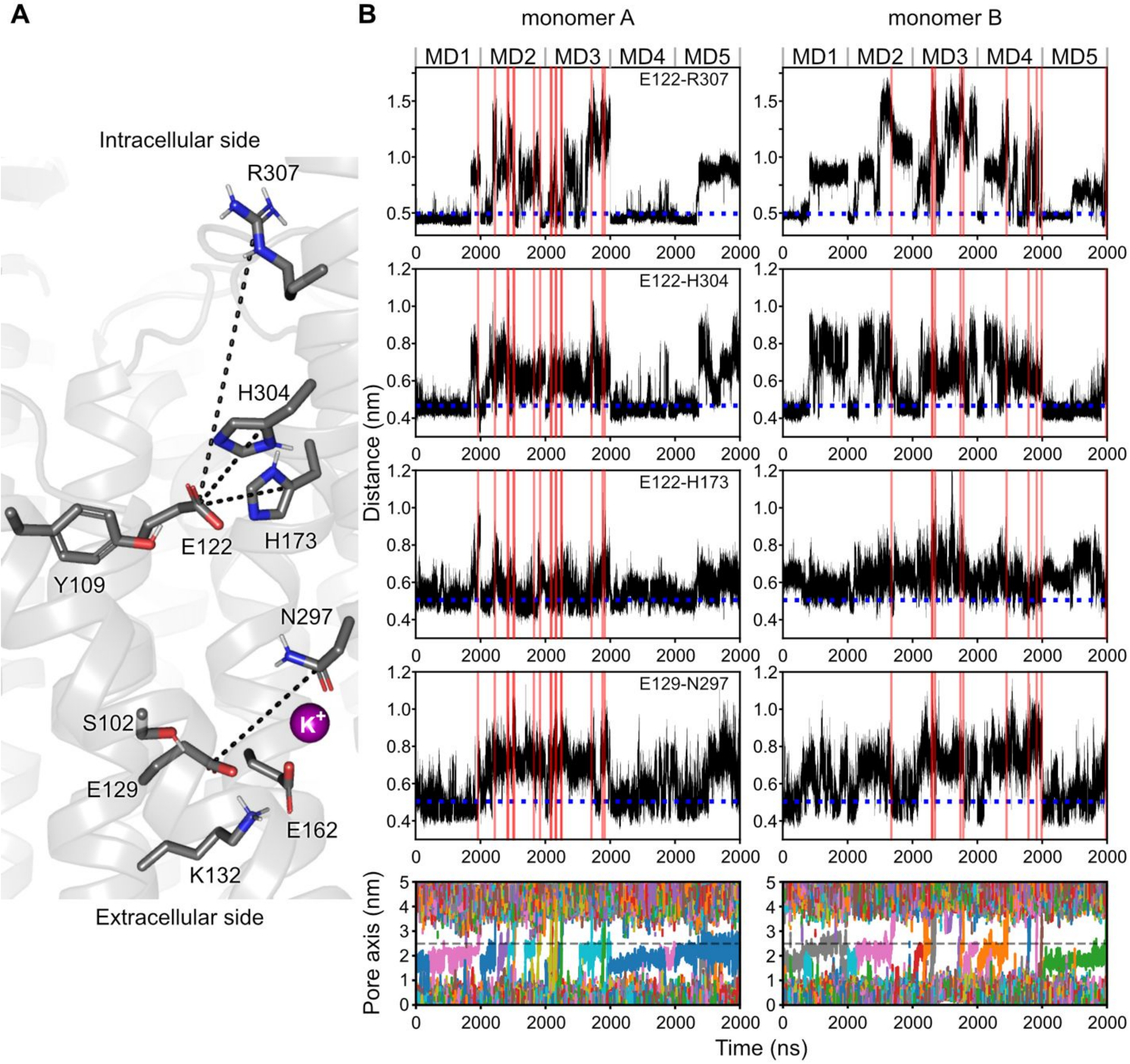

Supplementary Figure 6: Time evolution of residue distances during light-gating, and K^+ conduction events observed during the open state simulations. (**A**) Key residues considered for residue-pair distance measurements are depicted as stick models, and their calculated minimum distances are represented as dotted lines in the end snapshot of a 2-μs simulation of the C1C2 open state. (**B**) (from the first to the fourth rows) Time evolution of atomic distances of the key residues in the intracellular gate and central gate, and (in the fifth row) 1D ion tracking of K^+ within each pore with a radius of 2 nm and height of 5 nm, centered at each Cα of Ser102 in monomer A and B. (from the first to the fourth rows) The red lines indicate the corresponding inward K^+ permeation events passing through the central gate. The blue dotted lines represent the calculated minimal distances in our x-ray structure of M_{390}. The 2-μs simulations were replicated five times at 303 K with a 600 mM KCl concentration under a membrane potential of -389±27 mV.

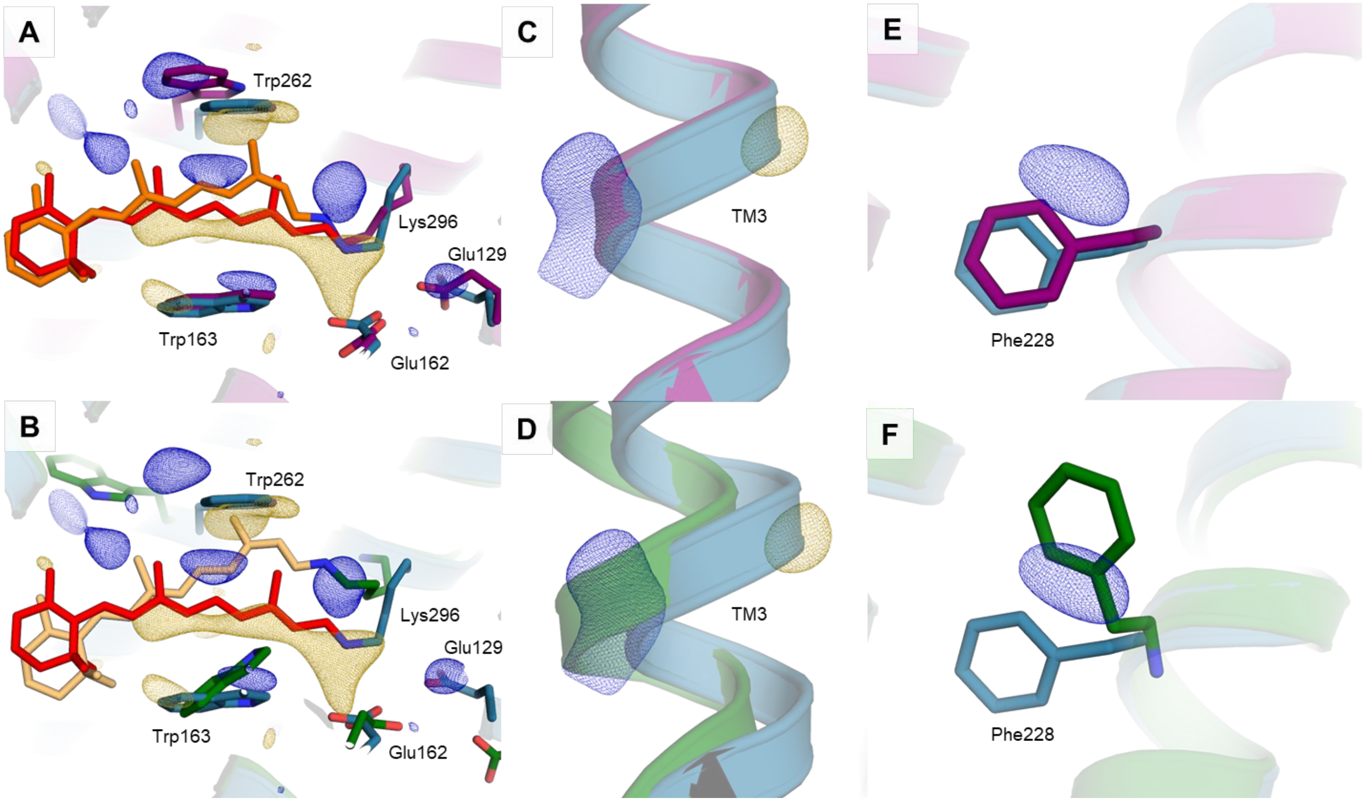

Supplementary Figure 7: Contributions of open state to light-activated data. (**A**) The refined structures (teal/red for dark and purple/orange for light-activated) explain the difference electron density map ((Fo(light)-Fo(dark), gold negative, blue positive, contoured at 3.5 sigma) well. (**B**) Even though the refined light-activated structure is the dominating state, an overlay of the difference electron density map with the simulated open state (green/lightorange) suggests that both intermediates contribute and the open state can occur *in crystallo*. Further evidence can be found in the transmembrane helix 3 (**C** and **D**) and on Phe228 (**E** and **F**). These observations provide a cross-validation for the molecular dynamic simulations and open the possibility to resolve both states by time-resolved structural biology.

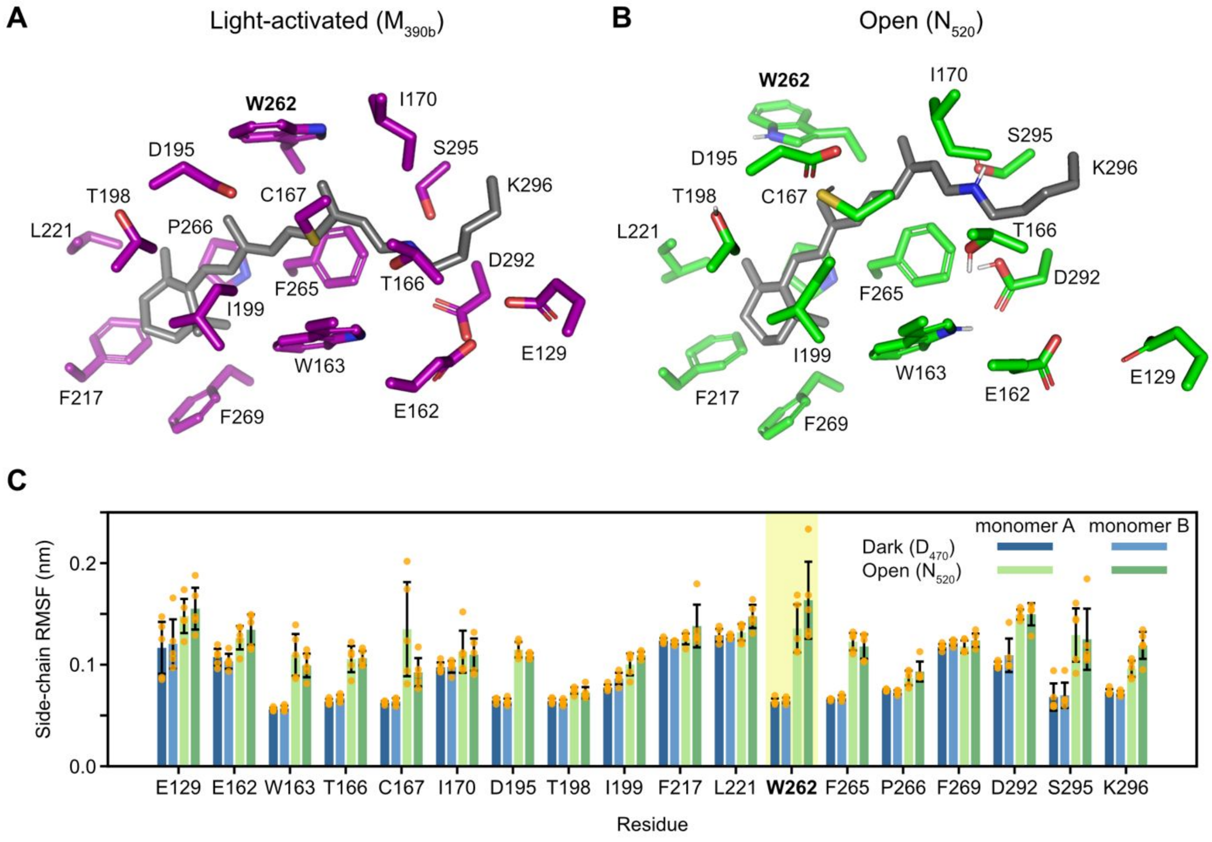

Supplementary Figure 8: Side-chain dynamics of the retinal binding site in C1C2. (**A**) Residues in the retinal binding site within 4.5 Å of the retinal moiety in the simulated M_{390b} state were selected for the calculation of root mean square fluctuation (RMSF) as shown in C. (**B**) The corresponding residues in the open state. (**C**) Residue-wise RMSF of the side-chain in the retinal binding site was calculated for each monomer in both the dark and open states. The 2-µs simulations were replicated five times at 303 K with a 600 mM KCl concentration under a membrane potential of -503±30 mV for the dark state and -389±27 mV for the open state.

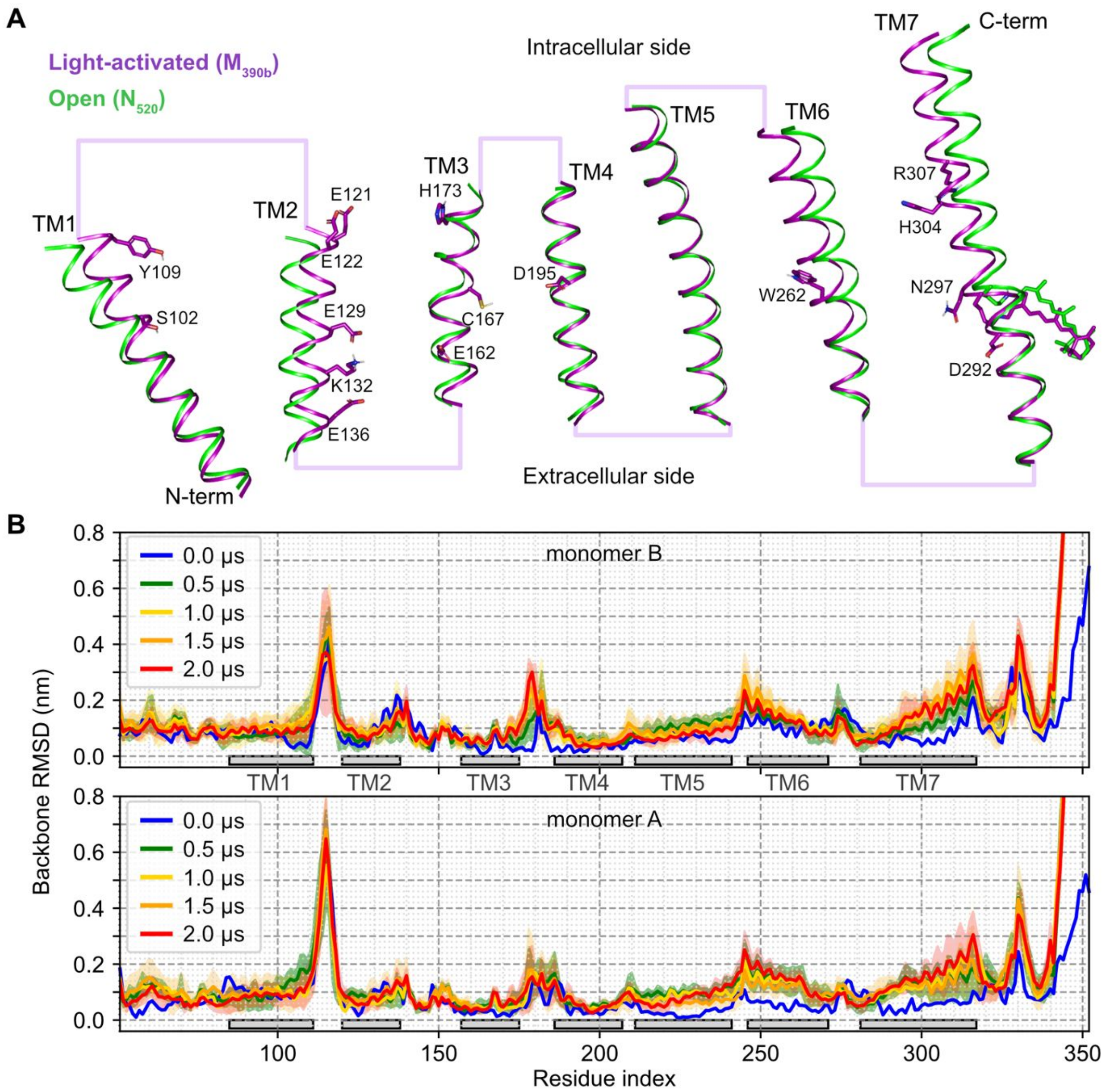

Supplementary Figure 9: Conformational transition from the light-activated state to the open state. (**A**) Cartoon representations of the transmembrane helices of the light-activated structure (purple, PDB entry 9GO2) and the end snapshot of an open state simulation replica at 2 μs. (**B**) Per-residue backbone root-mean-square deviations (RMSDs) of each monomer in the open state during production runs at 0, 0.5, 1, 1.5, and 2 μs. The reference structure for the RMSD calculation was set to the backbone of the light-activated structure. The 2-μs simulations of the open state were replicated five times at 303 K with a 600 mM KCl concentration under a membrane potential of -389±27 mV. The solid line and shading represent the mean and standard deviation of the RMSDs.

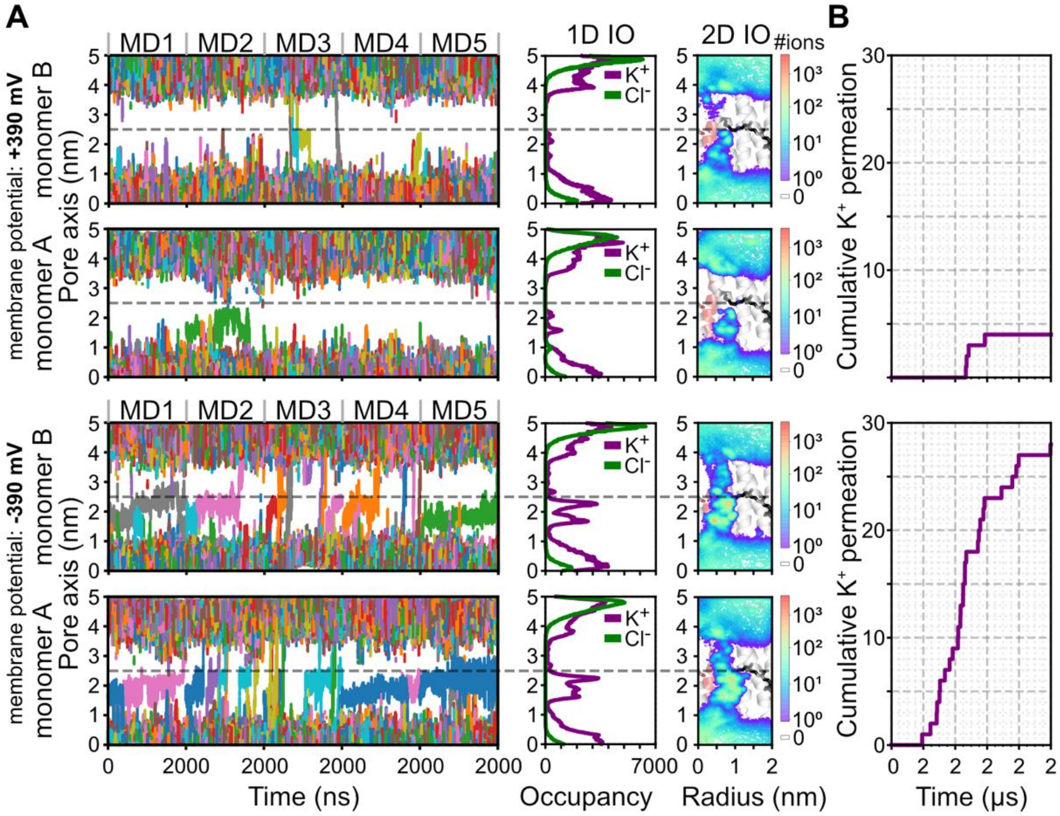

Supplementary Figure 10: Cation conduction through the open state of C1C2. (**A**) (**left**) Traces of K^+ in the pore of C1C2 with the central gate highlighted as a dashed grey line. (**middle**) Cumulative 1D ion occupancy along the pore axis. (**right**) 2D K^+ density within the pore region, mapped onto the open state C1C2, with protonated retinal Schiff base and Ser102 depicted as stick models. (**B**) Cumulative number of outward and inward K^+ permeation events passing through the central gate of C1C2 pore is shown in the upper and lower panels, respectively. (**A, B**) The 2-μs simulations were replicated five times at 303 K with a 600 mM KCl concentration.

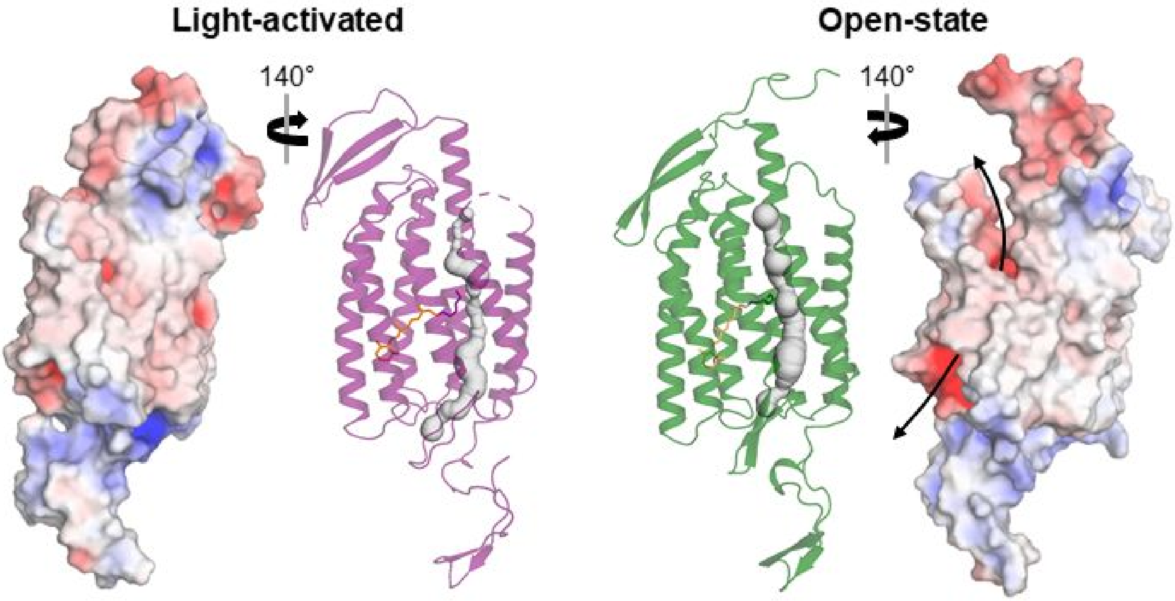

Supplementary Figure 11: Comparison of the charge distribution in the light-activated and open states of C1C2. The light-activated C1C2 structure (purple cartoon) and its electrostatic potential map (**left**) are compared with the representative open-state structure obtained by clustering from the MD simulation under a membrane potential of -390 mV and its electrostatic potential map (**right**), along with their putative ion-translocation channels (grey). In the electrostatic potential maps, positively and negatively charged regions are colored blue and red, respectively. The cation translocation pathway, indicated by a black arrow, is lined by negative charge supporting the selective flow of cations.

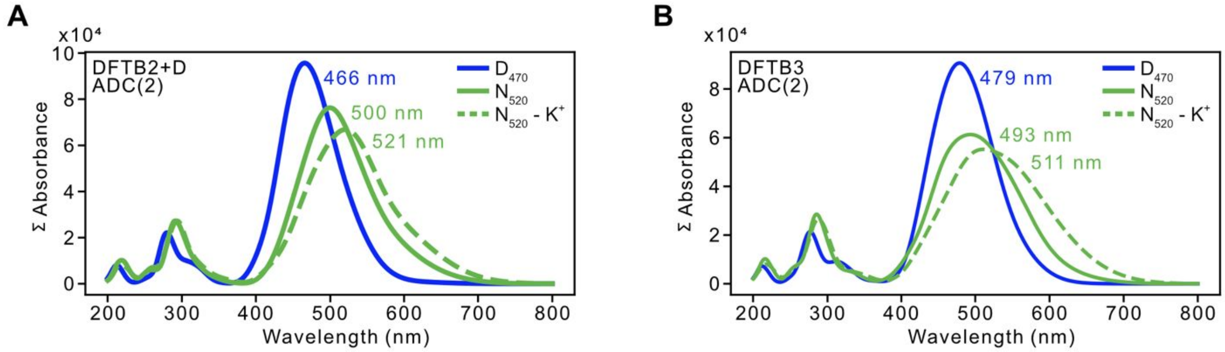

Supplementary Figure 12: Averaged vertical excitation energies of the dark and open (with and without K^+ in close vicinity of the retinal chromophore) states on RI-ADC(2)/cc-pVDZ level of theory, based on three QM/MM simulations runs for each state on (**A**) DFTB2+D and (**B**) DFTB3 level of theory.

Supplementary Movie 1: Potassium ion translocation via the open state. The MD trajectory from 850 ns to 875 ns of monomer A of the open state of C1C2 is shown. K^+, Cl^-, and water molecules are represented by purple, green, and cyan spheres, respectively. The key residues along the K^+ translocation pathway - (from the bottom to the top) Glu140, Glu136, Lys132, Glu162, Glu129, Asp292, Ser102, Asn297, Glu122, Glu121, His173, His304, and Arg307 - are depicted as stick models. For clarity, monomer B and POPC lipids are not shown, and only the K^+, Cl^-, and water molecules within 5 Å of the monomer A are displayed.

Supplementary Movie 2: Structural changes upon channel opening. This morph between the dark structure, the light-activated structure and the MD simulation using N_{520} protonation states illustrates the structural changes in the retinal binding pocket, the central gate and the intracellular gate upon channel formation.

Dataset	Dark	Light-activated
**Data Collection**		
Resolution (Å)	$C222_1$	$C222_1$
a, b, c (Å)	61.34, 141.70, 94.40	61.34, 141.70, 94.40
α, β, γ (°)	90, 90, 90	90, 90, 90
**Overall Statistics (High-Resolution Statistics)**		
Resolution (Å)	94.40-2.60 (2.70-2.60)	94.40-2.70 (2.80-2.70)
Indexed patterns	55249	43646
Indexing rate (%)	6.33	5.1
No. Reflections	13062 (1288)	11685 (1150)
Completeness (%)	100 (100)	100 (100)
Multiplicity	344.3 (139.2)	215.40 (33.2)
Rsplit (%)	7.93 (272.12)	9.4 (359.25)
CC*	0.999 (0.810)	0.999 (0.796)
<I/σ(I)>	7.39 (0.32)	6.37 (0.25)
**Anisotropy Corrected Statistics (High-Resolution Statistics)**		
Resolution (Å)	70.85-2.60 (2.69-2.60)	70.85-2.70 (2.80-2.70)
No. Reflections	8838 (1288)	7315 (72)
Completeness (%)	67.7 (10.3)	62.6 (6.3)
Multiplicity	415.94 (167.4)	348.47 (161.5)
Rsplit (%)	6.08 (87.08)	6.88 (84.77)
CC*	0.999 (0.849)	0.999 (0.75)
<I/σ(I)>	10.7 (1.05)	9.98 (1.16)
**Refinement**		
Resolution Range (Å)	70.85-2.59	70.85-2.70
No. Reflections	8,836	7312
R_{work} / R_{free}	0.2171 / 0.2696	0.2399 / 0.2682
**No. Atoms**		
Protein	2332	2327
Other	126	101
Water	13	4
**B Factors**		
Protein	54.6	42.8
Other	72.9	51.5
Water	40.8	31.9
**R.m.s. Deviations**		
Bond Lengths (Å)	0.005	0.004
Bond Angles (°)	0.919	0.810
**Ramachandran**		
Favored (%)	97.89	97.89
Allowed (%)	2.11	2.11
Outliers (%)	0.00	0.00
**PDB ID**	**9G01**	**9G02**

Supplementary Table 1: Crystallographic data and refinement statistics.

	Retinal Schiff base	Glu122	Glu129	Asp195	Asp292
**Dark state**	protonated	protonated	protonated	protonated	deprotonated
**Light-act.**	deprotonated	protonated	protonated	protonated	protonated
**Open state**	protonated	deprotonated	deprotonated	deprotonated	protonated

Supplementary Table 2: Protonation states used for MD simulations. Protonations for the dark-state, the early light-activated intermediate, and the open state were selected according to previous spectroscopic and electrophysiology experiments[34].

	N_{TOTAL}	$N_{POT/CL}$	N_{WAT}	N_{POPC}	*Dim*
**Dark-state**	261908	626/614	56824	528	10/10/25.6
**Light-act.**	262706	630/614	57054	530	10/10/25.6
**Open state**	262786	636/612	57082	530	10/10/25.6

Supplementary Table 3: Details of the CompEL simulation system. The simulations were conducted at 303 K with 600 mM KCl, and were individually replicated five times. N_{TOTAL}: the number of all atoms, N_{POT}/Cl_{CL}: the number of K^+ and Cl^-, N_{WAT}: the number of water molecules, N_{POPC}: the number of POPC, *Dim*: X/Y/Z box dimension (nm).

	Δt	t	k_{bb}	k_{sc}	k_{head}	$k_{torsion}$
Energy minimization	-	-	4000	2000	1000	1000
Equilibration 1	1	0.25	4000	2000	1000	1000
Equilibration 2	1	0.25	2000	1000	400	400
Equilibration 3	1	0.25	1000	500	400	200
Equilibration 4	2	1	500	200	200	200
Equilibration 5	2	1	200	50	40	100
Equilibration 6	2	1	50	0	0	0
Equilibration 7	2	100	0	0	0	0

Supplementary Table 4: Applied positional/dihedral angular restraints during energy minimization, equilibration simulations of C1C2. Time step: Δt (fs), individual simulation time: t (ns), force constant (kJ/mol/nm) for position/angle harmornic restraints of backbone heavy atoms (k_{bb}), side-chain heavy atoms (k_{sc}), lipid head group (k_{head}), and lipid chirality and cis double bond ($k_{torsion}$).

	Δq	t	V	N_I	G_I	N_O	G_O
**Dark-state**	4	1	343±36	0	0	0	0
	6	2	503±30	0	0	0	0
**Light-act.**	6	2	531±26	0	0	0	0
**Open state**	6	2	389±27	28	1.2±1.1	4	0.2±0.4

Supplementary Table 5: Ion conduction derived from the MD-based CompEL simulations. Δq: charge imbalance (e), V: membrane potential (mV), N_I: total inward permeations, G_I: inward conductance (pS), N_O: total outward permeations, G_O: outward conductance. All simulations were individually replicated five times at 303 K with ion concentration of 600 mM KCl.

References

(1) Kato, H. E.; Zhang, F.; Yizhar, O.; Ramakrishnan, C.; Nishizawa, T.; Hirata, K.; Ito, J.; Aita, Y.; Tsukazaki, T.; Hayashi, S.; et al. Crystal structure of the channelrhodopsin light-gated cation channel. *Nature* **2012**, *482* (7385), 369-U115.
(2) Scheib, U.; Broser, M.; Constantin, O. M.; Yang, S.; Gao, S.; Mukherjee, S.; Stehfest, K.; Nagel, G.; Gee, C. E.; Hegemann, P. Rhodopsin-cyclases for photocontrol of cGMP/cAMP and 2.3 A structure of the adenylyl cyclase domain. *Nat Commun* **2018**, *9* (1), 2046.
(3) Weinert, T.; Skopintsev, P.; James, D.; Dworkowski, F.; Panepucci, E.; Kekilli, D.; Furrer, A.; Brünle, S.; Mous, S.; Ozerov, D.; et al. Proton uptake mechanism in bacteriorhodopsin captured by serial synchrotron crystallography. *Science* **2019**, *365* (6448), 61-65.
(4) Weierstall, U.; James, D.; Wang, C.; White, T. A.; Wang, D.; Liu, W.; Spence, J. C.; Bruce Doak, R.; Nelson, G.; Fromme, P.; et al. Lipidic cubic phase injector facilitates membrane protein serial femtosecond crystallography. *Nat Commun* **2014**, *5*, 3309.
(5) White, T. A.; Kirian, R. A.; Martin, A. V.; Aquila, A.; Nass, K.; Barty, A.; Chapman, H. N. CrystFEL: a software suite for snapshot serial crystallography. *Journal of Applied Crystallography* **2012**, *45*, 335-341.
(6) Tickle, I. J., Flensburg, C., Keller, P., Paciorek, W., Sharff, A., Vonrhein, C., Bricogne, G. . *STARANISO*. Cambridge, United Kingdom: Global Phasing Ltd., 2018.
(7) De Zitter, E.; Coquelle, N.; Oeser, P.; Barends, T. R. M.; Colletier, J. P. Xtrapol8 enables automatic elucidation of low-occupancy intermediate-states in crystallographic studies. *Commun Biol* **2022**, *5* (1), 640.
(8) Adams, P. D.; Grosse-Kunstleve, R. W.; Hung, L. W.; Ioerger, T. R.; McCoy, A. J.; Moriarty, N. W.; Read, R. J.; Sacchettini, J. C.; Sauter, N. K.; Terwilliger, T. C. PHENIX: building new software for automated crystallographic structure determination. *Acta Crystallogr D Biol Crystallogr* **2002**, *58* (Pt 11), 1948-1954.
(9) Emsley, P.; Cowtan, K. Coot: model-building tools for molecular graphics. *Acta Crystallogr D Biol Crystallogr* **2004**, *60* (Pt 12 Pt 1), 2126-2132.
(10) Stourac, J.; Vavra, O.; Kokkonen, P.; Filipovic, J.; Pinto, G.; Brezovsky, J.; Damborsky, J.; Bednar, D. Caver Web 1.0: identification of tunnels and channels in proteins and analysis of ligand transport. *Nucleic Acids Res* **2019**, *47* (W1), W414-W422.
(11) Jo, S.; Kim, T.; Iyer, V. G.; Im, W. CHARMM-GUI: a web-based graphical user interface for CHARMM. *J Comput Chem* **2008**, *29* (11), 1859-1865.
(12) Sali, A.; Blundell, T. L. Comparative protein modelling by satisfaction of spatial restraints. *J Mol Biol* **1993**, *234* (3), 779-815.
(13) Jorgensen, W. L.; Chandrasekhar, J.; Madura, J. D.; Impey, R. W.; Klein, M. L. Comparison of Simple Potential Functions for Simulating Liquid Water. *J Chem Phys* **1983**, *79* (2), 926-935.
(14) (a) Tajkhorshid, E.; Paizs, B.; Suhai, S. Conformational effects on the proton affinity of the Schiff base in bacteriorhodopsin: A density functional study. *J Phys Chem B* **1997**, *101* (40), 8021-8028. (b) Tajkhorshid, E.; Suhai, S. Influence of the methyl groups on the structure, charge distribution, and proton affinity of the retinal Schiff base. *J Phys Chem B* **1999**, *103* (26), 5581-5590. (c) Tajkhorshid, E.; Baudry, J.; Schulten, K.; Suhai, S. Molecular dynamics study of the nature and origin of retinal's twisted structure in bacteriorhodopsin. *Biophys J* **2000**, *78* (2), 683-693.
(15) Huang, J.; Rauscher, S.; Nawrocki, G.; Ran, T.; Feig, M.; de Groot, B. L.; Grubmüller, H.; MacKerell, A. D. CHARMM36m: an improved force field for folded and intrinsically disordered proteins. *Nat Methods* **2017**, *14* (1), 71-73.
(16) Bussi, G.; Donadio, D.; Parrinello, M. Canonical sampling through velocity rescaling. *J Chem Phys* **2007**, *126* (1).

(17) Berendsen, H. J. C.; Postma, J. P. M.; Vangunsteren, W. F.; Dinola, A.; Haak, J. R. Molecular-Dynamics with Coupling to an External Bath. *J Chem Phys* **1984**, *81* (8), 3684-3690.

(18) Darden, T.; York, D.; Pedersen, L. Particle Mesh Ewald - an N.Log(N) Method for Ewald Sums in Large Systems. *J Chem Phys* **1993**, *98* (12), 10089-10092.
(19) Hess, B.; Bekker, H.; Berendsen, H. J. C.; Fraaije, J. G. E. M. LINCS: A linear constraint solver for molecular simulations. *Journal of Computational Chemistry* **1997**, *18* (12), 1463-1472.
(20) Kutzner, C.; Grubmüller, H.; de Groot, B. L.; Zachariae, U. Computational Electrophysiology: The Molecular Dynamics of Ion Channel Permeation and Selectivity in Atomistic Detail. *Biophys J* **2011**, *101* (4), 809-817.
(21) Abraham, M. J.; Murtola, T.; Schulz, R.; Páll, S.; Smith, J. C.; Hess, B.; Lindahl, E. GROMACS: High performance molecular simulations through multi-level parallelism from laptops to supercomputers. *SoftwareX* **2015**, *1-2*, 19-25.
(22) Michaud-Agrawal, N.; Denning, E. J.; Woolf, T. B.; Beckstein, O. Software News and Updates MDAnalysis: A Toolkit for the Analysis of Molecular Dynamics Simulations. *Journal of Computational Chemistry* **2011**, *32* (10), 2319-2327.
(23) Hunter, J. D. Matplotlib: A 2D graphics environment. *Comput Sci Eng* **2007**, *9* (3), 90-95.
(24) Virtanen, P.; Gommers, R.; Oliphant, T. E.; Haberland, M.; Reddy, T.; Cournapeau, D.; Burovski, E.; Peterson, P.; Weckesser, W.; Bright, J.; et al. SciPy 1.0: fundamental algorithms for scientific computing in Python. *Nat Methods* **2020**, *17* (3), 261-272.
(25) Harris, C. R.; Millman, K. J.; van der Walt, S. J.; Gommers, R.; Virtanen, P.; Cournapeau, D.; Wieser, E.; Taylor, J.; Berg, S.; Smith, N. J.; et al. Array programming with NumPy. *Nature* **2020**, *585* (7825), 357-362.
(26) Humphrey, W.; Dalke, A.; Schulten, K. VMD: Visual molecular dynamics. *J Mol Graph Model* **1996**, *14* (1), 33-38.
(27) D.A. Case, H. M. A., K. Belfon, I.Y. Ben-Shalom, S.R. Brozell, D.S. Cerutti, T.E. Cheatham, III, G.A. Cisneros, V.W.D. Cruzeiro, T.A. Darden, R.E. Duke, G. Giambasu, M.K. Gilson, H. Gohlke, A.W. Goetz, R. Harris, S. Izadi, S.A. Izmailov, C. Jin, K. Kasavajhala, M.C. Kaymak, E. King, A. Kovalenko, T. Kurtzman, T.S. Lee, S. LeGrand, P. Li, C. Lin, J. Liu, T. Luchko, R. Luo, M. Machado, V. Man, M. Manathunga, K.M. Merz, Y. Miao, O. Mikhailovskii, G. Monard, H. Nguyen, K.A. O'Hearn, A. Onufriev, F. Pan, S. Pantano, R. Qi, A. Rahnamoun, D.R. Roe, A. Roitberg, C. Sagui, S. Schott-Verdugo, J. Shen, C.L. Simmerling, N.R. Skrynnikov, J. Smith, J. Swails, R.C. Walker, J. Wang, H. Wei, R.M. Wolf, X. Wu, Y. Xue, D.M. York, S. Zhao, and P.A. Kollman. *Amber 21*. 2021.
(28) (a) Gaus, M.; Cui, Q. A.; Elstner, M. DFTB3: Extension of the Self-Consistent-Charge Density-Functional Tight-Binding Method (SCC-DFTB). *J Chem Theory Comput* **2011**, *7* (4), 931-948. (b) Walker, R. C.; Crowley, M. F.; Case, D. A. The implementation of a fast and accurate QM/MM potential method in Amber. *Journal of Computational Chemistry* **2008**, *29* (7), 1019-1031. (c) Seabra, G. D.; Walker, R. C.; Elstner, M.; Case, D. A.; Roitberg, A. E. Implementation of the SCC-DFTB method for hybrid QM/MM simulations within the amber molecular dynamics package. *J Phys Chem A* **2007**, *111* (26), 5655-5664.
(29) (a) Gaus, M.; Lu, X. Y.; Elstner, M.; Cui, Q. Parameterization of DFTB3/3OB for Sulfur and Phosphorus for Chemical and Biological Applications. *J Chem Theory Comput* **2014**, *10* (4), 1518-1537. (b) Gaus, M.; Goez, A.; Elstner, M. Parametrization and Benchmark of DFTB3 for Organic Molecules. *J Chem Theory Comput* **2013**, *9* (1), 338-354. (c) Kubillus, M.; Kubar, T.; Gaus, M.; Rezác, J.; Elstner, M. Parameterization of the DFTB3 Method for Br, Ca, Cl, F, I, K, and Na in Organic and Biological Systems. *J Chem Theory Comput* **2015**, *11* (1), 332-342.
(30) Elstner, M.; Hobza, P.; Frauenheim, T.; Suhai, S.; Kaxiras, E. Hydrogen bonding and stacking interactions of nucleic acid base pairs: A density-functional-theory based treatment. *The Journal of Chemical Physics* **2001**, *114* (12), 5149-5155.
(31) Dunning, T. H. Gaussian basis sets for use in correlated molecular calculations. I. The atoms boron through neon and hydrogen. *The Journal of Chemical Physics* **1989**, *90*, 1007-1023.
(32) Oda, K.; Vierock, J.; Oishi, S.; Rodriguez-Rozada, S.; Taniguchi, R.; Yamashita, K.; Wiegert, J. S.; Nishizawa, T.; Hegemann, P.; Nureki, O. Crystal structure of the red light-activated channelrhodopsin Chrimson. *Nat Commun* **2018**, *9* (1), 3949.

(33) Nogly, P.; Weinert, T.; James, D.; Carbajo, S.; Ozerov, D.; Furrer, A.; Gashi, D.; Borin, V.; Skopintsev, P.; Jaeger, K.; et al. Retinal isomerization in bacteriorhodopsin captured by a femtosecond x-ray laser. *Science* **2018**, *361* (6398).
(34) (a) Kuhne, J.; Eisenhauer, K.; Ritter, E.; Hegemann, P.; Gerwert, K.; Bartl, F. Early Formation of the Ion-Conducting Pore in Channelrhodopsin-2. *Angew Chem Int Edit* **2015**, *54* (16), 4953-4957. (b) Takemoto, M.; Kato, H. E.; Koyama, M.; Ito, J.; Kamiya, M.; Hayashi, S.; Maturana, A. D.; Deisseroth, K.; Ishitani, R.; Nureki, O. Molecular Dynamics of Channelrhodopsin at the Early Stages of Channel Opening. *Plos One* **2015**, *10* (6). (c) Shibata, K.; Oda, K.; Nishizawa, T.; Hazama, Y.; Ono, R.; Takaramoto, S.; Bagherzadeh, R.; Yaw, H.; Nureki, O.; Inoue, K.; et al. Twisting and Protonation of Retinal Chromophore Regulate Channel Gating of Channelrhodopsin C1C2. *J Am Chem Soc* **2023**, *145* (19), 10779-10789. (d) Watanabe, H. C.; Welke, K.; Sindhikara, D. J.; Hegemann, P.; Elstner, M. Towards an Understanding of Channelrhodopsin Function: Simulations Lead to Novel Insights of the Channel Mechanism. *J Mol Biol* **2013**, *425* (10), 1795-1814. (e) Kaufmann, J. C. D.; Krause, B. S.; Adam, S.; Ritter, E.; Schapiro, I.; Hegemann, P.; Bartl, F. J. Modulation of Light Energy Transfer from Chromophore to Protein in the Channelrhodopsin ReaChR. *Biophys J* **2020**, *119* (3), 705-716. (f) Lórenz-Fonfría, V. A.; Bamann, C.; Resler, T.; Schlesinger, R.; Bamberg, E.; Heberle, J. Temporal evolution of helix hydration in a light-gated ion channel correlates with ion conductance. *P Natl Acad Sci USA* **2015**, *112* (43), E5796-E5804.
